# Heart–Lung Interactions in Combined Distributive Shock and ARDS: Applied Cardiopulmonary Physiology at the Bedside

**DOI:** 10.3390/jcm14217844

**Published:** 2025-11-05

**Authors:** Athanasios Chalkias, Konstantina Katsifa, Stavroula Amanetopoulou, Georgios Karapiperis, Christos Tountas, Nikoleta Ntalarizou, Athanasios Prekates, Paraskevi Tselioti

**Affiliations:** 1 Institute for Translational Medicine and Therapeutics, Perelman School of Medicine, University of Pennsylvania, Philadelphia, PA 19104-5158, USA; 2 OUTCOMES RESEARCH Consortium^®^, Houston, TX 77030, USA; 3 Department of Critical Care Medicine, Tzaneio General Hospital, 18536 Piraeus, Greece; 4 Department of Cardiology, Sismanogleio General Hospital, 15126 Athens, Greece

**Keywords:** heart–lung interactions, cardiopulmonary interactions, cardiorespiratory interactions, distributive shock, septic shock, ARDS, applied cardiopulmonary physiology, critical care medicine, intensive care medicine

## Abstract

Distributive shock and acute respiratory distress syndrome (ARDS) are syndromes of profound pathophysiological complexity, each independently associated with high morbidity and mortality. When coexistent, they create a state of synergistic cardiopulmonary failure where conventional, protocolized management approaches are often insufficient. This review synthesizes current mechanistic insights into heart–lung interactions in distributive shock with ARDS, highlighting the central role of right ventricular–pulmonary arterial coupling and the dual impact of altered lung mechanics and vascular dysregulation. We examine the distinct hemodynamic implications of pulmonary versus extrapulmonary ARDS phenotypes, including their divergent effects on transpulmonary pressure, venous return, and right ventricular afterload, and emphasize the clinical relevance of mixed phenotypes. Advanced monitoring modalities—esophageal manometry, echocardiography, and, in select cases, pulmonary artery catheterization—are presented as essential tools for dynamic phenotyping and individualized titration of ventilatory and hemodynamic strategies. Building on these principles, we outline phenotype-directed approaches to ventilation, fluid and vasoactive therapy, and adjunctive interventions such as proning and extracorporeal support. Finally, we discuss knowledge gaps and future directions, underscoring the need for integrative technologies and phenotype-stratified trials to refine precision management. The nuanced integration of cardiopulmonary physiology into bedside decision-making represents a paradigm shift toward individualized, physiology-guided care for this high-risk population.

## 1. Introduction

Distributive shock, most commonly septic in origin, and acute respiratory distress syndrome (ARDS) are formidable syndromes that individually confer substantial morbidity and mortality in the intensive care unit (ICU). When they co-occur, they create a state of extreme physiological jeopardy where the insults are not merely additive but profoundly synergistic and mechanistic [1]. Contemporary data continue to report high fatality rates for this combined condition, often exceeding 40–50% in multicenter cohorts [2,3], particularly when complicated by refractory vasoplegia or acute cor pulmonale. The management of these patients epitomizes the nuance of applied critical care physiology, demanding that the clinician act as a real-time physiologist at the bedside.

Historically, management strategies have evolved from a focus on normalizing individual physiological variables to a more integrated, organ-protective philosophy. Early goal-directed therapy protocols for sepsis aimed to optimize specific hemodynamic targets. Similarly, the landmark ARMA trial established low tidal volume ventilation as the standard of care for ARDS [4], shifting the focus to mitigating ventilator-induced lung injury. However, a one-size-fits-all approach has proven inadequate for the intricate and highly variable pathophysiology of shock with ARDS [5]. The routine use of invasive tools like the pulmonary artery (PA) catheter (PAC), for instance, failed to show a survival benefit in broad populations [6], leading to a decline in its use but also highlighting the need for more targeted application in select, complex cases where specific physiological questions must be answered.

This evolution has led to the current era, which emphasizes personalized medicine rooted in a deep mechanistic understanding. The cornerstone of this approach is recognizing that positive-pressure ventilation, while lifesaving, is a potent hemodynamic intervention. Its cyclic and tonic effects on intrathoracic pressure dynamically influence venous return, biventricular loading conditions, and ventricular interdependence on a beat-to-beat basis. Consequently, seemingly minor adjustments to ventilator settings or vasoactive drug infusions can precipitate disproportionate—often catastrophic— hemodynamic consequences, frequently culminating in acute right ventricular (RV) failure [7,8]. Appreciating these intricate interactions is essential to avoid iatrogenic harm and tailor therapy to the individual patient’s unique physiology.

This review revisits the fundamental mechanisms of heart–lung interactions in combined distributive shock and ARDS, emphasizing the pivotal role of hemodynamic phenotyping in optimizing therapeutic strategies that concurrently preserve pulmonary function and RV performance.

## 2. The Intertwined Pathophysiology

The coexistence of distributive shock and ARDS creates a vicious cycle of organ injury. The systemic inflammation of sepsis drives endothelial injury, glycocalyx degradation, and microvascular thrombosis—processes that are amplified within the delicate pulmonary circulation. This shared pathology is then subjected to the complex and often opposing mechanical forces of positive-pressure ventilation, which can either sustain or collapse cardiovascular stability depending on the underlying mechanics.

### 2.1. Altered Lung Mechanics and the Concept of Injurious Power

Alveolar mechanics in ARDS are defined by profound heterogeneity. The lung transforms into a patchwork of collapsed, fluid-filled dependent regions and relatively preserved, aerated non-dependent regions. This gives rise to the “baby lung” concept, where the functional, ventilated lung is much smaller than the anatomical lung. Dependent units undergo cyclic atelectasis (recruitment and de-recruitment with each breath), generating shear stress and inflammation (atelectrauma), while non-dependent units are susceptible to overdistension (volutrauma), particularly as positive end-expiratory pressure (PEEP) is increased. This heterogeneity is the basis for two critical concepts in modern ventilator management, the driving pressure (ΔP) and mechanical power (MP).

Driving pressure, defined as plateau pressure minus PEEP, represents the cyclic strain applied to the lung parenchyma. A landmark post hoc analysis revealed that ΔP, not tidal volume or PEEP alone, was the variable most strongly associated with survival in ARDS [9]. Physiologically, it is the strongest ventilator-related predictor of acute RV failure, as the cyclic change in transpulmonary pressure (P_TP_ = Paw − Ppl) dictates the degree of alveolar capillary compression during inspiration, a major contributor to the pulsatile afterload faced by the right ventricle [10].

The concept of MP integrates multiple injurious components (i.e., tidal volume, respiratory rate, ΔP, and inspiratory flow) into a single variable representing the energy transferred from the ventilator to the respiratory system per minute [11]. High MP has been linked to both lung injury and RV dysfunction [12]. It highlights that a seemingly “protective” strategy with low tidal volume can still be injurious if the respiratory rate is excessively high. The ongoing challenge is normalizing MP to the size of the aerated “baby lung,” as the same power delivered to a smaller functional lung is undoubtedly more damaging.

### 2.2. The Dysregulated Systemic and Pulmonary Vasculature

**Figure 1:**
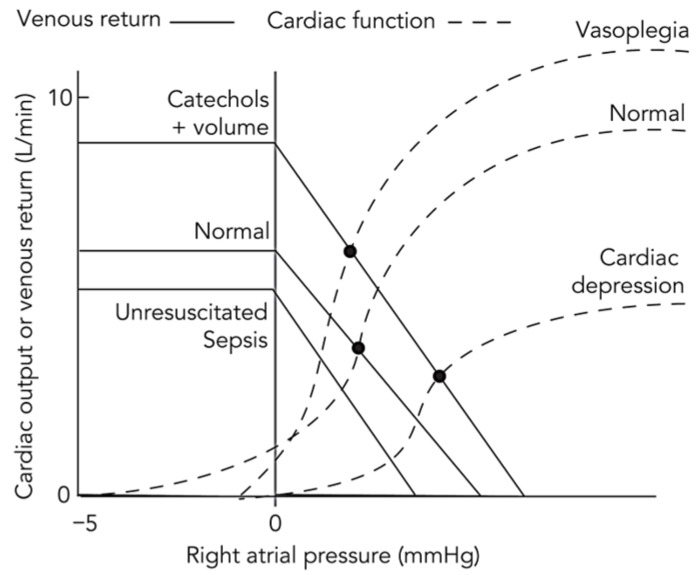
Conceptual representation of the Guyton–Frank–Starling model across physiological and pathological circulatory states. This schematic illustrates the integrated interaction between the venous return (Guyton) curve and the cardiac function (Frank–Starling) curve under varying hemodynamic conditions. The horizontal axis represents right atrial pressure, an index of cardiac filling, and the vertical axis represents either venous return or cardiac output, which are equal at steady state. Their intersection defines the equilibrium point of the circulation. In the normal state, balanced venous tone and mean systemic filling pressure maintain an adequate pressure gradient for venous return and an appropriate equilibrium cardiac output. In distributive shock, e.g., resuscitated sepsis, systemic vasodilation and capillary leak increase venous capacitance and reduce mean systemic filling pressure, shifting the venous return curve downward and rightward and lowering equilibrium cardiac output despite preserved myocardial contractility. Following volume expansion and catecholamine therapy, increased stressed volume and enhanced contractility restore venous return and elevate the cardiac function curve, shifting the equilibrium upward and leftward to reflect improved output at lower right atrial pressure. In vasoplegia after resuscitation, persistent vascular smooth muscle relaxation again depresses the venous return curve, limiting output despite preserved contractility. In sepsis with cardiac depression, myocardial performance is impaired, the cardiac function curve flattens, and equilibrium output declines further, yielding a mixed distributive-cardiogenic phenotype. This integrated Guyton-Frank-Starling model unifies the hemodynamic evolution from normal physiology to circulatory failure and highlights the interdependence of vascular tone, venous return, and myocardial function. Adapted from reference [14] under the terms of the Creative Commons Attribution-NonCommercial-NoDerivatives 4.0 International License (CC BY-NC-ND 4.0).

The vascular tone in both the systemic and pulmonary circuits becomes profoundly dysregulated during distributive shock. The release of vasodilatory mediators such as nitric oxide and prostaglandins causes marked systemic vasodilation, reducing systemic vascular resistance (SVR) and greatly increasing venous capacitance. This results in a state of relative hypovolemia. Within the framework of Guyton’s model of circulatory equilibrium, this widespread vasodilation—along with capillary leak—lowers the mean systemic filling pressure (P_MSF_), which represents the upstream pressure driving venous return (Figure 1) [13,14]. Conceptually, P_MSF_ is the theoretical pressure throughout the circulatory system if the heart were to stop, reflecting the elastic recoil of the vasculature acting on the contained blood volume. Venous return to the heart depends on the pressure gradient between this upstream P_MSF_ and the downstream right atrial pressure (P_RA_). As P_MSF_ falls, this gradient (P_MSF_ − P_RA_) diminishes, making the patient profoundly preload-dependent.

**Figure 2:**
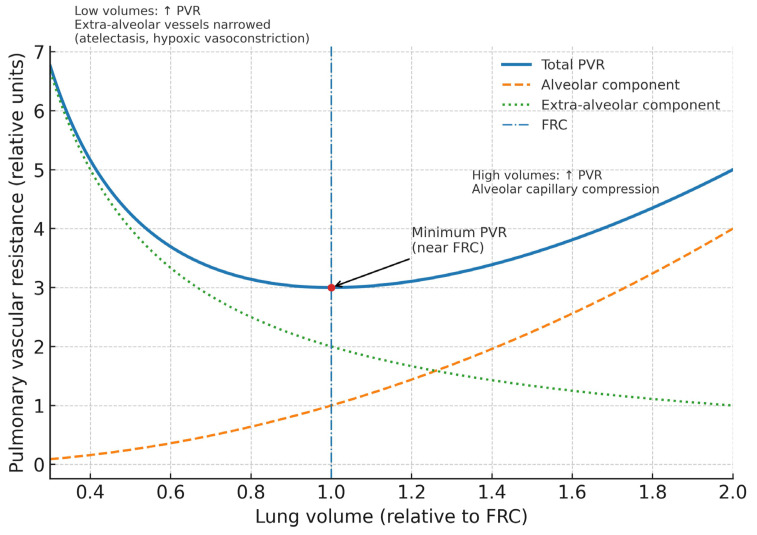
Relationship between lung volume and pulmonary vascular resistance in ARDS. The total pulmonary vascular resistance (solid line) follows a U-shaped curve, with a minimum near functional residual capacity (dash-dotted line). At low lung volumes, extra-alveolar vessels narrow due to atelectasis and hypoxic vasoconstriction, resulting in increased resistance (dotted line). At high lung volumes, alveolar capillaries are compressed, similarly elevating pulmonary vascular resistance (dashed line). The interplay of these opposing forces determines the nadir of pulmonary vascular resistance near functional residual capacity, which has important implications for ventilatory and hemodynamic management in ARDS. PVR, pulmonary vascular resistance; FRC, functional residual capacity.

In stark contrast, the pulmonary circulation becomes a high-resistance, high-impedance circuit, and the relationship between lung volume and pulmonary vascular resistance (PVR) is described by a U-shaped curve [15,16]. Pulmonary vascular resistance is minimized near the normal functional residual capacity. At low lung volumes, atelectasis and vessel tortuosity increase resistance in extra-alveolar vessels, a situation exacerbated by hypoxic pulmonary vasoconstriction, while at high lung volumes, the distended alveoli stretch and compress the delicate intra-alveolar capillaries, markedly increasing PVR (Figure 2). This mechanical compression is compounded by intrinsic vascular pathology: widespread endothelial injury, formation of microthrombi, and a dysregulated inflammatory milieu blunt the physiological hypoxic pulmonary vasoconstriction response in the most consolidated lung regions. This has the disastrous effect of increasing perfusion to non-ventilated, shunt-producing lung units, which worsens systemic hypoxemia while other vascular segments undergo profound vasoconstriction, thereby further increasing overall PVR.

## 3. ARDS Phenotypes: The Cornerstone of Hemodynamic Management

A pivotal advance in managing ARDS hemodynamics is the deconstruction of the syndrome into distinct phenotypes based on the primary site of mechanical derangement. The classic distinction between pulmonary ARDS (ARDSp) and extrapulmonary ARDS (ARDSexp) is not merely etiological; it has profound, predictable, and often opposite implications for cardiopulmonary interactions under positive-pressure ventilation [17,18,19,20,21]. The mechanical behavior of the respiratory system is governed by the relationship between the compliance of the lung (C_L_) and the chest wall (C_CW_). The change in pleural pressure (Ppl) during a passive mechanical breath is determined not by total respiratory system compliance, but almost exclusively by C_CW_, according to the fundamental equation: ΔPpl = ΔVolume/C_CW_ [17,22]. This simple but profound relationship dictates the hemodynamic fate of the patient.

### 3.1. Pulmonary ARDS

Arising from direct insults such as pneumonia or aspiration, this phenotype is characterized by dense alveolar consolidation and a marked reduction in C_L_, while C_CW_ is often preserved and remains relatively high. Consequently, most of the applied airway pressure is transmitted across the stiff lung parenchyma, generating a dangerously elevated P_TP_ [23,24]. Although alveolar pressure rises substantially in this setting, the low parenchymal compliance typical of the “stiff lung” limits the degree to which this pressure is transmitted to the pleural space. As a result, venous return tends to remain relatively preserved despite the severe increase in P_TP_—a distinction that underscores the dissociation between alveolar stress and intrathoracic pressure transmission in this phenotype. This markedly high P_TP_ compresses the pulmonary microvasculature, leading to a sharp rise in RV afterload.

**Figure 3:**
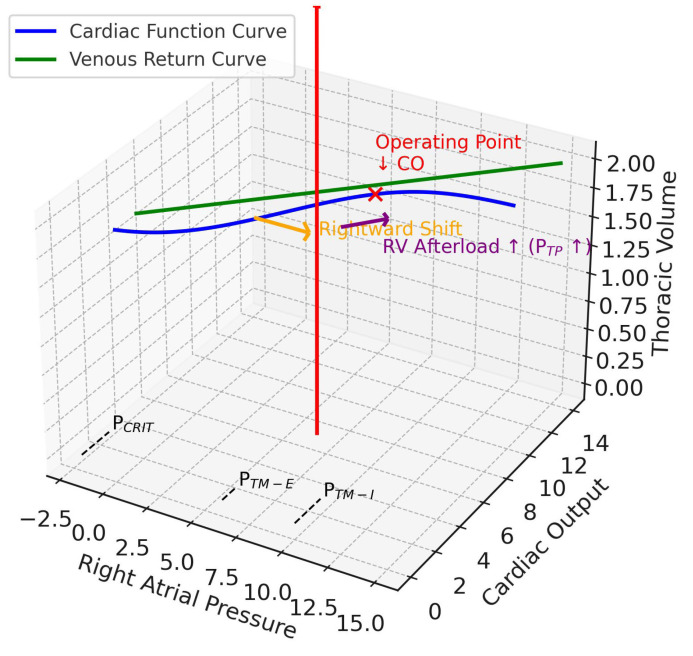
Pulmonary ARDS. This 3D diagram integrates the Guyton venous return and cardiac function curves (x–y plane) with the Campbell diagram (x–z plane). The z-axis is thoracic volume (i.e., a surrogate volume axis combining C_CW_ and C_L_), with the red arrow indicating tidal volume delivered during positive pressure ventilation. In ARDSp, reduced lung compliance shifts the respiratory system compliance so that transpulmonary pressure rises. This pushes the cardiac function curve rightward (orange arrow, preload effect) and flattens its slope (purple arrow, afterload effect), reflecting increased right ventricular afterload. The operating point (red x mark) shifts downward, showing reduced cardiac output at higher right atrial pressures. Together, these changes impair right ventricular filling, elevate afterload, and promote RV dilatation in pulmonary ARDS. CO, cardiac output; RV, right ventricular; P_TP_, transpulmonary pressure; P_CRIT_, critical collapse pressure, marked on the venous return curve at the right atrial pressure axis intercept; P_TM-E_, expiratory right atrial transmural pressure, closer to baseline; P_TM-I_, inspiratory right atrial transmural pressure, elevated on the right atrial pressure axis.

In this “stiff lung” phenotype, the patient is highly susceptible to afterload-dominant RV failure. Importantly, it is the resultant increase in lung volume—rather than the elevation in P_TP_ per se—that accounts for many of the observed alterations in PVR. This distinction is crucial, as both alveolar overdistension and recruitment alter pulmonary vessel geometry, thereby increasing RV afterload and predisposing to circulatory failure. Because the change in Ppl is minimal, the direct effects of positive-pressure ventilation on venous return and left ventricular (LV) afterload are less pronounced. Hemodynamically, as illustrated in the conceptual Campbell-Guyton diagram, this manifests as a marked flattening of the cardiac function curve’s slope (reflecting increased afterload) with only a slight rightward shift (indicating minimal change in preload), ultimately producing a new low-flow equilibrium point (Figure 3) [17]. This mechanism explains the clinical picture of a patient with severe pneumonia who develops acute cor pulmonale and hemodynamic collapse in response to an increase in PEEP or tidal volume.

### 3.2. Extrapulmonary ARDS

Driven by systemic inflammation from sources such as pancreatitis or abdominal sepsis, this phenotype is characterized by interstitial edema and, crucially, a primary reduction in C_CW_ due to factors such as intra-abdominal hypertension, anasarca, or obesity. In the present review, the term “extrapulmonary ARDS” is used in its physiomechanical rather than purely etiologic sense. While traditionally defined as ARDS arising from a non-pulmonary insult (e.g., pancreatitis, trauma, or sepsis with a non-pulmonary source), here it denotes a mechanical phenotype in which reduced C_CW_ and elevated Ppl—often due to intra-abdominal hypertension, anasarca, or obesity—govern cardiopulmonary interactions. This mechanistic distinction highlights the hemodynamic consequences of pressure transmission from the abdomen and thoracic cage on venous return and RV loading.

**Figure 4:**
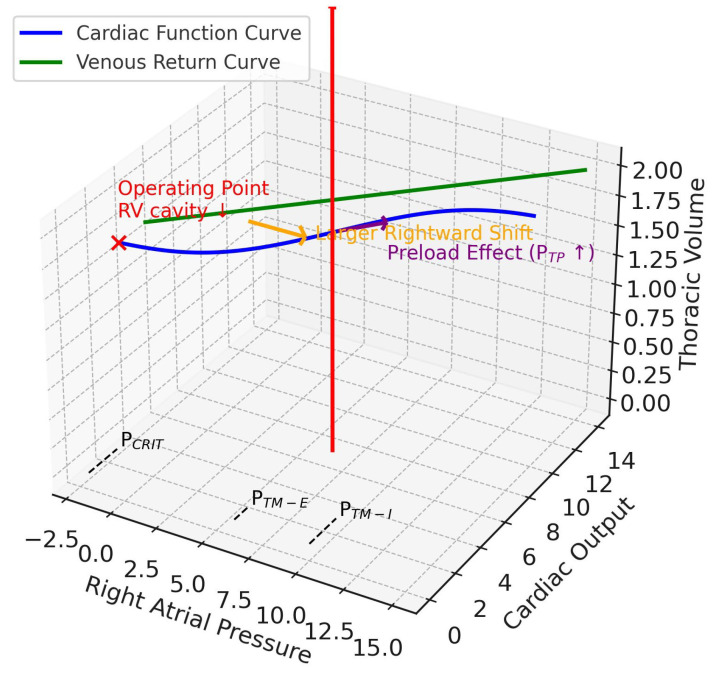
Extrapulmonary ARDS. This 3D diagram integrates the Guyton venous return and cardiac function curves (x–y plane) with the Campbell diagram (x–z plane). The z-axis is thoracic volume (i.e., a surrogate volume axis combining C_CW_ and C_L_), with the red arrow indicating tidal volume delivered during positive pressure ventilation. In ARDSexp, reduced respiratory system compliance is driven mainly by reduced chest wall compliance. With positive pressure ventilation, pleural pressure rises more prominently, producing a larger rightward shift of the cardiac function curve (orange arrow), while its slope remains relatively preserved (since transpulmonary pressure does not rise as much). Thus, preload is affected more strongly than afterload. The operating point (red x mark) shifts rightward along the venous return curve, with reduced transmural right atrial pressure and relatively preserved cardiac output. The net effect is reduced right ventricular preload and afterload, leading to RV cavity shrinkage (contrasting with ARDSp). RV, right ventricular; P_TP_, transpulmonary pressure; P_CRIT_, critical collapse pressure, marked on the venous return curve at the right atrial pressure axis intercept; P_TM-E_, expiratory right atrial transmural pressure, closer to baseline; P_TM-I_, inspiratory right atrial transmural pressure, elevated on the right atrial pressure axis.

In this “stiff chest wall” scenario, a mechanical breath generates a large increase in Ppl because C_CW_ is low. This has a cascade of hemodynamic effects. Firstly, the high Ppl is transmitted directly to the intrathoracic great veins and right atrium, dramatically impeding the pressure gradient for venous return and causing preload-dominant circulatory compromise. However, because Ppl rises substantially, the P_TP_ for any given airway pressure is much lower (but still higher than that in normal conditions), mitigating the compressive effect on the pulmonary vasculature and thus the increase in RV afterload [17]. Simultaneously, the high intrathoracic pressure envelops the left ventricle, decreasing its transmural pressure and effectively reducing LV afterload, which can be beneficial in patients with pre-existing heart failure or septic cardiomyopathy [20,25,26,27,28,29,30,31,32]. The Guytonian representation shows a significant rightward shift of the cardiac function curve (preload reduction) but a far less pronounced flattening of its slope (less severe afterload increase), leading to a complex new equilibrium (Figure 4) [17].

### 3.3. Mixed/Alternative ARDS Phenotype

In clinical practice, a significant proportion of patients display characteristics of both phenotypes, particularly after dual insults (e.g., pancreatitis complicated by a ventilator-associated pneumonia) or during prolonged ICU stays. These patients present a profound therapeutic dilemma. They experience a dual mechanical burden: a stiff chest wall that impedes venous return (reducing preload) and stiff lungs that increase RV afterload. Any attempt to recruit the consolidated lung with higher PEEP or ΔP risks further increasing RV afterload due to elevated Ptp, while simultaneously worsening preload reduction as a result of the inevitable rise in Ppl. This phenotype highlights the critical limitations of global measurements such as plateau pressure and emphasizes the need for advanced monitoring to differentiate respiratory mechanics from cardiovascular dynamics, enabling clinicians to navigate these competing physiological stresses (Table 1).

## 4. The Right Ventricle: From Physiology to Failure

The right ventricle, a thin-walled, volume-sensitive chamber adapted to a low-impedance pulmonary circuit, is the central nexus of heart–lung interactions in distributive shock with ARDS. Its unique crescentic geometry and thin free wall render it vulnerable to acute increases in afterload.

### 4.1. Ventricular-Arterial Coupling

A sophisticated understanding of RV function requires moving beyond simple descriptive terms to a rigorous biophysical framework known as ventricular–arterial coupling. This concept evaluates how efficiently energy is transferred from the ventricle to the arterial system and is defined by the ratio of end-systolic elastance (Ees) to effective arterial elastance (Ea) [33,34].

**Figure 5:**
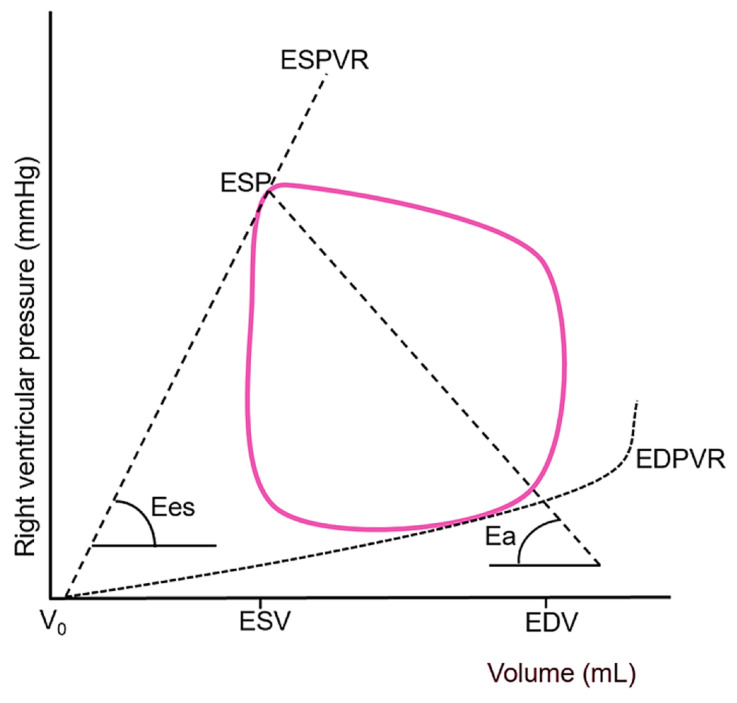
Representative right ventricular pressure–volume loop demonstrating the derivation of end-systolic elastance and effective arterial elastance, which together define the efficiency of energy transfer between the ventricle and the pulmonary circulation (ventricular–arterial coupling). The end-systolic pressure–volume relationship (solid red line) is constructed by connecting the end-systolic points of multiple pressure–volume loops obtained under varying loading conditions, with its slope representing Ees, i.e., the load-independent index of intrinsic myocardial contractility. A steeper end-systolic pressure–volume relationship indicates enhanced contractile performance, whereas a shallower slope, as typically observed in septic cardiomyopathy. Effective arterial elastance (dashed line) serves as an integrative measure of total RV afterload encompassing both resistive (steady) and pulsatile (reactive) components. ESPVR, end-systolic pressure-volume relationship; ESP, end-systolic pressure; Ees, end-systolic elastance; Ea, effective arterial elastance; EDPVR, end-diastolic pressure-volume relationship; V_0_, hypothetical uncompressed ventricular volume; ESV, end-systolic volume; EDV, end-diastolic volume. Adapted from reference [37] under the terms of the Creative Commons Attribution (CC BY) license.

End-systolic elastance represents the most accurate, load-independent measure of intrinsic myocardial contractility, or inotropy [35,36,37]. It is defined by the slope of the end-systolic pressure-volume relationship (ESPVR)—a line connecting the end-systolic points of multiple pressure-volume loops generated under different loading conditions. A steeper slope reflects greater contractile strength. Conceptually, Ees can be envisioned as the ventricular “stiffness” or contractile force at the end of systole, independent of both preload and afterload (Figure 5).

A healthy ventricle behaves like a resilient spring, capable of generating substantial tension (pressure) even with minimal deformation (volume change). In septic shock, however, RV Ees is often markedly reduced due to the multifactorial mechanisms of sepsis-induced cardiomyopathy, including cytokine-mediated myocardial depression, mitochondrial dysfunction, and impaired β-adrenergic signaling [38]. This reduction results in a flatter ESPVR, significantly impairing the right ventricle’s ability to adapt to increased afterload.

Effective arterial elastance is a comprehensive measure of the total afterload imposed on the ventricle, calculated as the ratio of end-systolic pressure to stroke volume (ESP/SV) [39]. It encompasses all factors that oppose ventricular ejection and consists of both a steady (resistive) and a pulsatile (reactive) component. The steady component is the PVR, which, as discussed, is markedly elevated in ARDS due to a combination of mechanical compression, microthrombi, endothelial swelling, and vasoconstriction. The pulsatile component, often underappreciated, is also critically important to the right ventricle and includes PA compliance (Cpa) and wave reflection.

Under normal conditions, the pulmonary circulation is highly compliant. However, in ARDS, it becomes stiff, so a given stroke volume produces a much larger pulse pressure, thereby increasing the pulsatile workload of the right ventricle [40]. Furthermore, in this stiff, high-resistance system, pressure waves generated during systole are reflected back from the periphery earlier, arriving in late systole and directly opposing forward ejection, which further augments afterload. Therefore, Ea provides a more comprehensive assessment of ventricular afterload than PVR alone [41].

### 4.2. Ventricular-Arterial Uncoupling

Optimal energy transfer from the right ventricle to the pulmonary circulation occurs when the Ees/Ea ratio is approximately 1.5–2.0 [42]. A ratio falling below 1.0 signifies RV-PA uncoupling, a state of profound bioenergetic inefficiency where the ventricle can no longer effectively eject blood against its load, leading to a rapid decline in stroke work and eventual failure. The patient with septic shock and ARDS presents a textbook physiological model of impending uncoupling: sepsis actively suppresses RV Ees while ARDS and mechanical ventilation dramatically increase PA Ea [43,44,45,46].

The transition from a coupled to an uncoupled state is often precipitous, governed by a vicious, self-amplifying feedback loop. The right ventricle initially adapts via the Frank-Starling mechanism and homeometric adaptation (Anrep effect), but these are finite and compromised by sepsis. Once the right ventricle dilates beyond a critical point, the Law of Laplace dictates that its wall stress increases exponentially. This elevated wall stress has catastrophic consequences: it directly impairs contractility by overstretching sarcomeres, dramatically increases myocardial oxygen demand, and simultaneously reduces myocardial oxygen supply by compressing the right coronary artery during diastole, all in the setting of systemic hypotension. The uncoupled right ventricle consumes excessive oxygen for minimal effective work, spiraling into ischemia, energetic failure, and bioenergetic collapse. This manifests clinically as a falling cardiac output, a rising central venous pressure (CVP), and progressive systemic hypoperfusion refractory to conventional therapy.

### 4.3. Ventricular Interdependence and the Pericardial Constraint

The hemodynamic fate of the left ventricle is inextricably linked to the right ventricle through both diastolic and systolic ventricular interdependence, amplified by the relatively non-compliant pericardium [47,48]. As the pressure- and volume-overloaded right ventricle dilates, the shared interventricular septum flattens and bows leftward. This septal shift directly impairs LV diastolic filling by decreasing its effective compliance, leading to a fall in LV end-diastolic volume and stroke volume, irrespective of systemic venous return [49]. Additionally, systolic ventricular interaction, whereby LV contraction normally contributes up to 40% of RV pressure generation, is also compromised [50]. This interdependence is a critical mechanism by which a primary pulmonary and right-sided pathology translates directly into systemic circulatory failure.

## 5. Advanced Monitoring: From Data to Physiological Insight

Effective management hinges on moving beyond static filling pressures and embracing advanced monitoring to characterize the underlying phenotype and track RV function in real-time.

### 5.1. Pulmonary Artery Catheter

While not for routine use, the PAC can be invaluable in the most complex cases of refractory shock or suspected RV failure where specific physiological questions must be answered [51,52]. It is the only tool that allows for direct measurement of the PAP waveform and calculation of PVR, the transpulmonary gradient (TPG), and the diastolic pulmonary gradient (DPG). A DPG > 7 mmHg, for example, is highly suggestive of a pre-capillary (vasoreactive/obstructive) component of pulmonary hypertension that might be amenable to targeted therapy with inhaled pulmonary vasodilators. Continuous monitoring of mixed venous oxygen saturation (SvO_2_) provides a real-time assessment of the global adequacy of oxygen delivery to consumption; a falling SvO_2_ is often the earliest and most sensitive sign of impending RV decompensation and circulatory failure.

Importantly, because Peso monitoring is not available in many ICUs, the PAC offers a pragmatic and widely accessible alternative for advanced hemodynamic phenotyping. By continuously tracking P_RA_, mean PAP (mPAP), pulmonary capillary wedge pressure (PCWP), cardiac output, and SvO_2_, the clinician can differentiate whether circulatory failure is dominated by preload limitation, excessive RV afterload, or biventricular myocardial depression. Derived indices such as PVR, Cpa, and the DPG add mechanistic resolution by quantifying the steady, pulsatile, and microvascular components of RV afterload.

For practical bedside interpretation, specific hemodynamic patterns derived from the PAC can clarify the dominant physiological mechanism. For instance, a rising mPAP accompanied by a normal PCWP and a falling SvO_2_ typically indicates escalating RV afterload, most often seen in ARDSp or when excessive P_TP_ cause alveolar overdistension. In contrast, simultaneous elevation of P_RA_ and PCWP together with a reduced cardiac output is more consistent with biventricular myocardial depression or excessive levels of PEEP impairing venous return. Conversely, when both P_RA_ and PCWP are low while cardiac output is high and SVR is decreased, the hemodynamic picture is dominated by vasoplegia, characteristic of distributive shock physiology. These interpretive frameworks allow clinicians to titrate ventilator and vasoactive settings according to the underlying pathophysiology rather than fixed numeric targets.

In this context, PAC-derived data guide targeted intervention—whether to reduce ΔP and PEEP to alleviate RV afterload, initiate inotropes such as dobutamine or milrinone to augment contractility, add selective pulmonary vasodilators to improve coupling, or titrate vasopressors to restore coronary perfusion pressure. Continuous SvO_2_ and thermodilution-derived cardiac output trends often signal evolving RV–PA uncoupling before echocardiographic changes become evident, offering a valuable early warning of impending hemodynamic collapse.

The practical integration of PAC data into bedside management is summarized in Table 2.

### 5.2. Critical Care Echocardiography

Echocardiography is vital for the modern management of these patients. It provides a direct, non-invasive assessment of RV size and function [e.g., RV/LV ratio, tricuspid annular plane systolic excursion (TAPSE), RV S’ wave velocity, fractional area change (FAC), evidence of pressure overload (septal flattening, D-shaped left ventricle)], and can be used to estimate PA pressure (PAP) [9,51,53,54,55]. Its most critical role is in guiding dynamic interventions. A “fluid challenge,” for instance, should ideally be performed under direct echo guidance, assessing for a positive response (e.g., >15% increase in left ventricular outflow tract velocity time integral (LVOT VTI) while simultaneously ensuring there is no detrimental effect on the right ventricle (i.e., no worsening RV dilation)]. This helps to distinguish patients who are “fluid responsive” from those who are merely “fluid tolerant”.

### 5.3. Esophageal Manometry

This is the only clinical tool that allows for the bedside partitioning of respiratory system mechanics by providing a surrogate for Ppl [56,57,58,59,60]. It remains indispensable for calculating the true P_TP_ and the right atrial transmural pressure (P_RATM_= P_RA_ − Ppl), which is the effective filling pressure of the right ventricle. This allows for more precise PEEP titration to prevent both alveolar collapse (maintaining a slightly positive end-expiratory P_TP_) and overdistension (limiting end-inspiratory P_TP_ < 25 cmH_2_O). Furthermore, in patients who are not deeply sedated, esophageal pressure (Peso) can quantify patient respiratory effort, which is crucial for diagnosing and managing patient self-inflicted lung injury [61,62]. Although it is considered the reference standard for separating lung and chest-wall mechanics, esophageal manometry requires specific expertise and is not readily available in all ICUs. Its successful application demands appropriate training, equipment, and interpretation within a physiology-guided monitoring framework.

### 5.4. Emerging Monitoring Technologies

Three other tools deserve mention for their potential to refine ventilator management. The Stress Index, derived from the shape of the pressure-time waveform during constant flow ventilation, can be a specific bedside sign of overdistension (Stress Index > 1) versus tidal recruitment (Stress Index < 1) [63]. Electrical impedance tomography is a non-invasive, radiation-free monitoring tool that provides real-time, breath-by-breath images of regional ventilation distribution, allowing for PEEP titration to achieve the most homogenous lung inflation, minimizing both atelectasis and overdistension simultaneously [64].

Dynamic arterial elastance (Eadyn) has emerged as another functional indicator for assessing vasopressor responsiveness. Calculated as the ratio of pulse pressure variation to stroke volume variation [Eadyn = SVV/PPV], Eadyn provides a real-time index of the arterial load. It essentially quantifies the dynamic relationship between the heart and the arterial system, predicting how mean arterial pressure (MAP) will respond to changes in cardiac output. This is particularly valuable in distributive shock, where hypotension can be caused by both hypovolemia and vasodilation. A low Eadyn (e.g., <0.8) suggests high arterial compliance (vasodilation). In this scenario, even if the patient is fluid-responsive (high PPV/SVV), increasing stroke volume with fluids is unlikely to significantly raise blood pressure. This indicates a primary need for vasopressors to restore arterial tone. A high Eadyn (e.g., >1.0) implies that the arterial tone is preserved. Therefore, an increase in stroke volume following a fluid challenge will likely translate into a meaningful increase in MAP. However, Eadyn is only reliable in patients who are fully sedated, passively ventilated with a stable tidal volume, and in a regular sinus rhythm.

## 6. Phenotype-Driven Management Principles

### 6.1. Hemodynamic Phenotypes in Distributive Shock with ARDS

Four recurring bedside patterns frame the hemodynamic management of distributive shock with ARDS:

(1) Predominant vasoplegia with preserved biventricular function: Mean arterial pressure is low with high cardiac output and impaired oxygen extraction. The principal mechanism is systemic vasodilation with preserved ventricular-arterial coupling. Therapy centers on norepinephrine as first-line and vasopressin in catecholamine-resistant states to restore stressed volume without raising PVR. Ventilation should avoid excessive P_TP_ to prevent secondary RV loading.
(2) Right ventricular-dominant dysfunction with ARDSp: Elevated P_TP_ and PVR lead to RV dilation, septal shift, and systemic hypotension. The central pathophysiology is afterload-dominant RV failure caused by alveolar overdistension and vascular compression. Management emphasizes RV unloading through reduction in ΔP, careful PEEP titration, and early prone positioning, which redistributes transpulmonary stress, recruits dorsal regions, and decreases Ea, thereby improving RV-PA coupling. Inhaled pulmonary vasodilators (nitric oxide or epoprostenol) and inodilators (dobutamine or milrinone) may be added if low-flow states persist despite optimized afterload. Vasopressin is preferred over α-agonists to optimize MAP and maintain pulmonary/coronary perfusion without increasing PAP.
(3) Biventricular myocardial depression (distributive-cardiogenic overlap): This mixed phenotype exhibits global systolic dysfunction, often sepsis-induced, with depressed RV Ees and impaired LV ejection. Treatment balances vasopressors and inotropes while minimizing RV afterload. Avoid hypercapnia and acidosis, which elevate PVR and worsen coupling. Prone positioning and selective pulmonary vasodilation can simultaneously augment oxygenation and reduce RV wall stress.
(4) Mixed or evolving phenotypes: Many patients transition between preload- and afterload-dominant states due to changes in lung mechanics, abdominal pressure, or infection pattern. Prone positioning can both unload the right ventricle and, if abdominal compliance is limited, transiently reduce venous return; therefore, continuous monitoring of preload markers and RV size is required. Management demands dynamic adjustment rather than fixed protocols, alternating between strategies that optimize venous return (lower PEEP, relieve intra-abdominal pressure) and those that reduce RV afterload (limit ΔP, use prone or inhaled vasodilators) depending on the prevailing physiology.

Of note, prone positioning serves not merely as an oxygenation adjunct but as a targeted hemodynamic intervention that enhances RV-PA coupling, mitigates adverse ventricular interdependence, and promotes a more homogeneous distribution of P_TP_. Table 3 summarizes the corresponding vasoactive strategies tailored to each hemodynamic phenotype.

### 6.2. Ventilatory Strategies: Phenotype-Directed

Positive-pressure ventilation modulates venous return and ventricular loading conditions on a beat-to-beat basis. The rise in intrathoracic pressure during inspiration raises P_RA_, reducing the gradient for venous return; simultaneously, it lowers LV afterload by decreasing transmural systolic pressure. Positive end-expiratory pressure adds a steady-state increase in intrathoracic pressure that can either help or harm depending on the phenotype. In ARDSp with RV overload, high P_TP_ magnifies PVR and can precipitate acute cor pulmonale; in ARDSexp with high Ppl and RV underfilling, modest PEEP may stabilize the circulation by recruiting lung while not meaningfully increasing P_TP_, provided abdominal pressure is managed and total intrathoracic pressure is not excessive. Thus, the ventilator should be titrated as both a gas-exchange therapy and a hemodynamic intervention.

Ventilator management must pursue the dual goals of lung protection and RV protection. Low tidal volume ventilation (4–6 mL kg^−1^ predicted body weight) and limiting plateau pressure remain foundational, but phenotype and RV status determine the safe “ceiling” for ΔP. The optimal PEEP is one that maximizes alveolar recruitment without causing significant overdistension or hemodynamic compromise. In focal ARDS (often ARDSp), high PEEP is unlikely to recruit the consolidated lung and will preferentially overdistend healthier regions, worsening PVR and shunt [65]. In diffuse, recruitable ARDS (often ARDSexp), incremental PEEP can sequentially open collapsed lung units, decreasing overall PVR. A “PEEP trial” or recruitment-to-derecruitment maneuver should be performed with concurrent hemodynamic monitoring. Esophageal pressure-guided titration to achieve a positive end-expiratory P_TP_ (e.g., 0–2 cmH_2_O) while limiting end-inspiratory P_TP_ (<25 cmH_2_O) is the most physiologically sound approach [66].

Of note, an elevated arterial partial pressure of oxygen (PaO_2_) does not equate to improved tissue oxygenation if the intervention used to achieve it (e.g., excessive PEEP) reduces cardiac output. Current guidelines support targeting a PaO_2_ of 55–80 mmHg or an SpO_2_ of 88–95%. While trials like LOCO2 and ICU-ROX have shown mixed results on the benefits of conservative oxygenation, they suggest that a one-size-fits-all target is insufficient [67,68]. Lung-protective ventilation often necessitates permissive hypercapnia. While generally tolerated, severe hypercapnia (e.g., pH < 7.15–7.20) can lead to respiratory acidosis, which is a potent pulmonary vasoconstrictor, increases PA Ea, worsens RV function, and has direct negative inotropic effects on the myocardium [69].

Management principles for mixed phenotype ARDS require dynamic adjustment rather than fixed protocols. Ventilator settings should be individualized by combining ΔP targets, P_TP_ monitoring, and regional recruitment assessments. Positive end-expiratory pressure titration should account for both overdistension risk and potential alveolar reopening, possibly using a recruitment-to-inflation ratio or electrical impedance tomography if available. Monitoring frequency should be higher than in single-phenotype ARDS, as shifts between pulmonary and extrapulmonary–like behavior can occur within hours (Table 4).

For illustrative composite cases that exemplify these concepts in clinical practice, see Case Box.

### 6.3. Fluids, Vasopressors, and the Venous Side of the Circulation

In distributive states, venous capacitance expands and P_MSF_ falls. Modest fluid loading may be necessary early, but in the presence of ARDS the margin for fluid excess is narrow because interstitial edema worsens oxygenation and raises PVR. Also, a positive cumulative fluid balance after 24–48 h is strongly associated with worse outcomes. Therefore, fluid strategy should be conservative once perfusion is restored, using dynamic assessments (passive leg raise with echo, venous excess ultrasound, flow-based indices) rather than static pressures. The management can be conceptualized using a structured approach like the “Resuscitation, Optimization, Stabilization, Evacuation” (ROSE) model [70].

Standard dynamic measures like PPV and SVV are often unreliable in ARDS due to low tidal volumes, low C_L_, and critically, RV failure [71]. A “tidal volume challenge” can transiently improve their reliability [72], but the passive leg raise maneuver combined with real-time cardiac output monitoring remains the most robust predictor of fluid responsiveness [73]. The CVP, while heavily criticized as a measure of volume, remains a clinically important parameter as it provides an ongoing indication of the equilibrium between preload and the heart’s ability to handle it [74]; a rapid rise in CVP with a fluid challenge for minimal gain in cardiac output indicates the heart is on the flat portion of its Frank-Starling curve [58,75,76].

The choice and titration of vasoactive and inotropic agents must consider their effects on both the systemic and pulmonary circulations. By raising systemic pressure, vasopressors can improve coronary perfusion to the stressed right ventricle and may improve ventilation/perfusion (V/Q) matching. Norepinephrine is the first-choice vasopressor [77,78]. Its potent α_1_-adrenergic effects increase SVR and MAP, while its modest β_1_-effects support cardiac contractility. However, norepinephrine may increase PVR and RV afterload. Vasopressin, added to norepinephrine in refractory shock, acts on V_1_ receptors to cause systemic vasoconstriction while potentially sparing or even dilating the pulmonary circulation, making it a physiologically attractive choice in patients with RV failure [79,80]. Phenylephrine, a pure α_1_-agonist, should generally be avoided as it can significantly increase both PVR and SVR, worsening RV afterload without improving cardiac output [81].

Dobutamine is the inotrope of choice for septic myocardial dysfunction [77]. It primarily acts on β_1_-receptors to increase myocardial contractility (Ees) and stroke volume. However, by increasing cardiac output, it can worsen V/Q mismatch by overriding hypoxic pulmonary vasoconstriction and increasing shunt fraction, potentially impairing arterial oxygenation [82]. Milrinone, a phosphodiesterase-3 inhibitor, offers the theoretical advantage of being an “inodilator” (providing both inotropy and pulmonary vasodilation), but its long half-life and potential for profound systemic hypotension make it difficult to titrate in septic shock.

### 6.4. Adjunctive and Rescue Therapies

When conventional management fails, rescue therapies targeting the core physiological derangements are required. Proning, indicated for moderate-to-severe ARDS [PaO_2_/fraction of inspired oxygen (FiO_2_) < 150], dramatically improves survival [83]. It is associated with multiple favorable physiological effects: it recruits dorsal lung regions; makes P_TP_ more homogeneous; improves V/Q matching; and, crucially, unloads the right ventricle by reducing PA Ea and altering the gravitational orientation of the heart within the mediastinum to alleviate pericardial constraint [84].

Veno-venous (VV) extracorporeal membrane oxygenation (ECMO) can be a life-saving therapy for refractory hypoxemia [85,86]. A key benefit is its ability to facilitate “ultra-protective” or “rest” ventilation (e.g., ΔP near zero). This maximally rests the injured lung, reduces biotrauma, and profoundly unloads the right ventricle by minimizing airway pressures, making it arguably the most potent therapy for acute cor pulmonale driven by injurious ventilation [87]. Veno-arterial (VA) ECMO is a fundamentally different therapy reserved for refractory circulatory collapse due to irreversible RV or biventricular failure [88]. Extracorporeal carbon dioxide (CO_2_) Removal (ECCO_2_R) uses lower blood flows than ECMO and is an option for patients with intractable hypercapnic acidosis that limits the application of a lung-protective strategy. By managing CO_2_, ECCO_2_R allows for further reductions in tidal volume and ΔP, thereby protecting the lung and the right ventricle [89].

## 7. Special Considerations

### 7.1. Obesity and Intra-Abdominal Hypertension

In obesity, chest wall elastance is increased and airway pressure transmits more readily to pleural space; P_TP_ estimation becomes especially valuable to achieve alveolar recruitment without penalizing venous return [21]. In ARDSexp with intra-abdominal hypertension, venous return is impeded by raised abdominal venous pressures and diaphragmatic cephalad displacement that heightens Ppl [20]. Esophageal manometry reveals under-distension despite seemingly high plateau pressures. Management includes avoiding excessive PEEP, optimizing abdominal domain (nasogastric drainage, neuromuscular blockade for abdominal wall tone, percutaneous drainage or decompressive laparotomy when indicated), and prioritizing strategies that restore venous return (positioning, cautious fluids or venoconstrictors) while preventing overdistension.

### 7.2. Sedation, Analgesia, and Ventilator Synchrony

The precise management of sedation and analgesia constitutes a cornerstone—not a peripheral consideration—of lung- and RV-protective strategies. Patient–ventilator dyssynchrony, particularly when vigorous spontaneous inspiratory efforts are present, can fundamentally undermine the benefits of protective ventilation. Such patient-driven efforts may generate pronounced negative Ppl_s_ and occultly elevated P_TPs_, precipitating patient self-inflicted lung injury (P-SILI). The pathophysiology of P-SILI is mediated by pendelluft (i.e., intrapulmonary gas shifts from non-dependent to dependent lung regions during inspiration), regional overdistension, and repetitive alveolar recruitment–derecruitment, all of which exacerbate mechanical stress, amplify inflammatory cascades, and worsen pulmonary edema.

Achieving patient–ventilator synchrony is therefore paramount. Advanced monitoring techniques, particularly esophageal manometry, are invaluable for detecting injurious respiratory efforts by enabling estimation of P_TP_ and work of breathing [61,62]. Contemporary practice favors analgesia-first strategies, which prioritize optimal pain control to attenuate noxious stimuli and mitigate respiratory drive, in conjunction with judicious, titrated sedation to the minimal depth necessary for patient comfort and ventilator synchrony.

In cases of early, severe ARDS where dyssynchrony persists despite optimized analgesia and sedation, a brief course of intermittent or continuous neuromuscular blockade may be indicated to preserve authentic lung- and RV-protective ventilation. By abolishing spontaneous respiratory effort, neuromuscular blockade ensures precise delivery of the prescribed low tidal volume and ΔP, thereby minimizing MP and facilitating adjunctive interventions such as prone positioning and controlled permissive hypercapnia. Importantly, this profound sedation is inherently temporary. Sedation depth should be reassessed daily, with the objective of transitioning to lighter sedation as hemodynamic stability and gas exchange permit. This approach facilitates participation in spontaneous awakening and breathing trials (SBTs), which are essential for assessing weaning readiness and promoting early mobilization.

### 7.3. Weaning and Recovery

As the underlying drivers of distributive shock and ARDS resolve and the patient’s oxygenation and hemodynamics stabilize, the therapeutic goals pivot towards liberation from mechanical ventilation. This phase is fraught with its own physiological challenges, as the transition from positive-pressure to negative-pressure ventilation can unmask underlying cardiac dysfunction and precipitate derecruitment or RV decompensation. Therefore, the process must be systematic and patient-centered. Daily assessments of readiness to wean are crucial and should include objective evidence of clinical improvement, such as improving hemodynamics with minimal or no vasopressor support, decreasing requirements for FiO_2_ and PEEP, the presence of an adequate cough reflex, and sufficient neurological engagement. When a patient meets these criteria, an SBT should be conducted.

The settings for the SBT must be carefully chosen to approximate the physiological load the patient will experience post-extubation, typically involving low (or very low) levels of pressure support with minimal PEEP. Failure of an SBT may manifest as respiratory distress, but a significant proportion of failures are cardiovascular in origin. The switch to negative-pressure breathing increases venous return and left ventricular afterload, which can precipitate weaning-induced pulmonary edema in patients with underlying diastolic or systolic dysfunction [48,90].

In patients with a history of significant RV strain during their acute illness, the weaning process requires particular caution. A graduated approach, with a progressive reduction in ventilatory support rather than an abrupt SBT, may be necessary. Close hemodynamic monitoring, potentially including focused echocardiography during the weaning trial, can help identify early signs of RV decompensation, allowing clinicians to tailor the weaning strategy to prevent recurrent failure and promote successful liberation.

## 8. Quality Improvement and Future Directions

Outcome improvements in complex syndromes such as sepsis shock with ARDS depend on team coordination, shared mental models, and timely feedback. Implementing structured rounds that explicitly address lung and RV protection—for example, asking “What is the ΔP today? What is the Peso-estimated P_TP_? What do echo and gas exchange tell us about RV-PA coupling?”—helps anchor decision-making in shared physiologic targets. Simulation-based training in proning logistics, hemodynamic rescue, and ECMO cannulation reduces procedural errors and builds confidence across disciplines. Real-time dashboards that integrate ventilator data, echo metrics, and vasoactive doses can flag deterioration early and provide a common reference point for multidisciplinary teams. Embedding such tools into daily workflow fosters anticipation rather than reaction, allowing clinicians to intervene before irreversible cardiovascular failure or injurious ventilation occurs (Table 5). Dashboards also serve as an educational bridge, reinforcing physiology-based decision-making for trainees while ensuring alignment across critical care, cardiology, and perfusion services (Table 6 and Table 7).

Despite advances, several knowledge gaps persist. First, we lack pragmatic, bedside tools that continuously track RV-PA coupling and P_TP_. Second, although the etiological distinction between ARDSp and ARDSexp offered limited prognostic value in large clinical trials and was therefore excluded from the Berlin Definition, it remains a valuable conceptual framework for understanding their divergent underlying mechanics. However, clinical trials rarely stratify by ARDS phenotype or RV status, diluting potential signals of benefit from targeted interventions. Third, integration of abdominal pressure into ventilator titration and hemodynamic management remains underutilized outside specialized centers.

Future trials should prospectively stratify by ARDS phenotype, RV status (normal vs. uncoupled), and chest wall mechanics (normal vs. high Ppl). Endpoints must capture not only oxygenation but also hemodynamics (RV-PA coupling surrogates, need for rescue RV support) and patient-centered outcomes. Technology priorities include minimally invasive Ppl measurement, continuous RV strain monitoring, and decision-support algorithms that titrate PEEP/ΔP to preserve coupling while avoiding overdistension. Advances in wearable sensors and catheter-based technologies may eventually allow continuous, bedside assessment of chest wall mechanics and RV performance in a way that is both practical and scalable. Integrating these physiologic signals into machine-learning–driven platforms could enable adaptive ventilation strategies that respond in real time to shifts in preload, afterload, and lung mechanics.

Beyond the ICU, multicenter registries that harmonize physiologic and outcome data will be critical to validate phenotype-protective approaches and accelerate translation into practice. Ultimately, sustained quality improvement in this domain will hinge not only on technological innovation, but also on cultivating multidisciplinary expertise, standardized protocols, and robust feedback systems that reinforce long-term change.

## 9. Take-Home Messages for Clinical Practice

- **Prioritize accurate phenotypic characterization:** Precise identification of the ARDS phenotype forms the foundation for individualized ventilatory and hemodynamic management. Before modifying ventilator settings, determine whether the dominant limitation arises from the lung parenchyma (ARDSp) or the chest wall/extrapulmonary compartment (ARDSexp). In ARDSp, RV protection through minimization of the ΔP is paramount. In ARDSexp, hemodynamic compromise often results from Ppl and reduced venous return; management should therefore focus on relieving intra-abdominal hypertension and applying PEEP judiciously.
- **Use transpulmonary pressure to guide ventilation:** Transpulmonary pressure, rather than plateau pressure, should direct lung-protective ventilation to achieve optimal alveolar recruitment while avoiding overdistension and RV loading.
- **Driving pressure is the critical determinant:** Among ventilatory variables, ΔP demonstrates the strongest association with mortality and RV dysfunction. When ΔP exceeds 15 cmH_2_O, the clinical imperative is to reduce it—accepting permissive hypoxemia or hypercapnia if necessary—to safeguard both pulmonary and RV function.
- **Recognize the central role of the right ventricle:** The right ventricle is the physiological nexus linking pulmonary mechanics, oxygenation, and systemic perfusion. If hypotension develops following an increase in PEEP, acute RV failure should be suspected before assuming vasoplegia. Even a focused bedside echocardiographic assessment of RV size and function can provide critical diagnostic insight.
- **Employ early prone positioning for right ventricular protection:** In ARDSp with evidence of RV strain, prone positioning should be instituted early. Beyond improving oxygenation, proning serves as an effective RV unloading maneuver, reducing pulmonary vascular impedance and improving ventricular interdependence.
- **Integrate multimodal physiological monitoring:** Although esophageal manometry and echocardiography remain the most robust bedside tools for individualized titration of ventilation and hemodynamics, a multimodal assessment strategy incorporating dynamic/static surrogates (e.g., CVP trends, fluid responsiveness indices) and, when appropriate, invasive modalities such as the PAC may be warranted in complex cases.
- **Adopt a physiologically grounded hemodynamic strategy:** Once adequate tissue perfusion has been established, a conservative fluid management strategy should be adopted to minimize RV congestion and pulmonary edema. In the context of vasoplegia or persistent hypotension, particularly when RV afterload sensitivity or elevated pulmonary vascular resistance is evident, preferential use of vasopressin over catecholamines may be physiologically advantageous. This approach exemplifies a precision physiology framework, emphasizing the individualized modulation of preload, afterload, and vascular tone rather than reflexive fluid administration or adrenergic escalation.
- **Continuously reassess the physiological phenotype:** The coexistence of distributive shock and ARDS represents a state of exceptional pathophysiological complexity. An initially extrapulmonary (ARDSexp) phenotype -for example, secondary to pancreatitis- may evolve into a mixed or pulmonary-dominant (ARDSp) form following secondary pneumonia. Ongoing physiological reassessment is therefore imperative to ensure that ventilatory and hemodynamic interventions remain appropriately tailored to the patient’s dynamic pathophysiological state.

## 10. Conclusions

The management of patients with distributive shock and ARDS is the quintessence of applied critical care physiology, demanding a paradigm shift from one-size-fits-all protocols towards a nuanced, individualized approach rooted in a deep understanding of heart–lung interactions. Central to this endeavor is the precise delineation of the patient’s dynamic cardiopulmonary phenotype. This review has articulated a framework centered on the critical distinction between ARDSp and ARDSexp, demonstrating how the primary site of respiratory derangement dictates fundamentally different hemodynamic consequences in response to positive-pressure ventilation. Establishing such a framework is essential for advancing personalized management while simultaneously guiding research to elucidate pathophysiologic mechanisms and uncover treatment targets in critically ill patients.
**CASE BOX****A. Phenotype paradigms: Pulmonary ARDS** ***Case 1: ARDSp with afterload-dominant RV Failure*** Patient presentation: A 64-year-old male with a history of COPD is admitted with severe community-acquired pneumonia, rapidly progressing to septic shock and ARDS (PaO_2_/FiO_2_ of 105). Physiological data:Ventilator: Tidal volume (V_T_) 6 mL kg^−1^ PBW, PEEP 14 cmH_2_O, Pplat 30 cmH_2_O, ΔP 16 cmH_2_O.Hemodynamics: MAP 65 mmHg on norepinephrine, CVP rises from 12 to 18 mmHg over 2 h, lactate 4.2 mmol L^−1^.Monitoring: Bedside echocardiography reveals a severely dilated right ventricle (RV/LV ratio > 1.2), septal flattening throughout the cardiac cycle, and a tricuspid annular plane systolic excursion (TAPSE) of 11 mm.Clinical challenge: The patient’s hypoxemia prompted an increase in PEEP from 10 to 14 cmH_2_O. While oxygenation transiently improved, it was followed by worsening hypotension requiring an escalating dose of norepinephrine and a rising CVP, classic signs of acute cor pulmonale from excessive RV afterload. Management and rationale:Reduce RV afterload: The ΔP was immediately reduced to 13 cmH_2_O by lowering the tidal volume to 5.5 mL kg^−1^, accepting permissive hypercapnia (pH maintained >7.25). The high ΔP and PEEP were generating excessive P_TP_, compressing the pulmonary vasculature in a poorly recruitable lung. Adding vasopressin permitted a reduction in norepinephrine dose, which further decreased PVR.Optimize PEEP: PEEP was de-escalated to 10 cmH_2_O, as the consolidated lung was unlikely to recruit further and the high PEEP was primarily causing overdistension of healthier lung zones and increasing PVR.Initiate prone positioning: The patient was placed in the prone position. This therapy is known to improve V/Q matching and, critically, unload the right ventricle by homogenizing P_TPs_ and altering the heart’s position in the mediastinum.Targeted vasodilation: Inhaled epoprostenol was initiated as a rescue therapy to selectively dilate the pulmonary vasculature without causing systemic hypotension, directly addressing the high PVR.Outcome: Within 4 h of these interventions, the CVP decreased to 13 mmHg, the norepinephrine requirement was reduced by half, and the RV size on echo began to normalize. Teaching point: In ARDSp with poor recruitability, escalating PEEP to treat hypoxemia can precipitate life-threatening RV failure. The therapeutic priority must shift to RV protection by minimizing ΔP and considering therapies like proning that unload the right heart. ***Case 2: ARDSp with patient self-inflicted lung injury (P-SILI)*** Patient Presentation: A 52-year-old female is intubated for ARDS secondary to aspiration pneumonitis. She is on lung-protective settings but remains tachypneic and appears to be in respiratory distress. Physiological data:Ventilator: V_T_ 6 mL/kg PBW, RR set at 22 min^−1^, Total RR 35 min^−1^, PEEP 12 cmH_2_O, Pplat 28 cmH_2_O.Hemodynamics: Tachycardic (125 bpm), MAP 70 mmHg on low-dose norepinephrine.Monitoring: Esophageal manometry is placed. During a patient-triggered breath, the Peso swings from +12 cmH_2_O at end-expiration to −8 cmH_2_O, generating a peak P_TP_ (=Paw − Ppl) of 36 cmH_2_O.Clinical challenge: Despite seemingly “protective” ventilator settings, the patient’s vigorous, uncontrolled inspiratory efforts are generating massive, injurious P_TPs_. This patient P-SILI is likely worsening her lung injury and contributing to RV strain through high cyclic afterload. Management and rationale:Control respiratory drive: An analgesia-first strategy was optimized, followed by an increase in sedation via propofol infusion to blunt the central respiratory drive.Ablate injurious effort: When dyssynchrony persisted, a continuous infusion of cisatracurium (neuromuscular blockade) was initiated for 24 h. This was critical to gain full control of the ventilator, eliminate P-SILI, and ensure the delivery of a truly protective V_T_ and ΔP.Peso-guided ventilation: With the patient passive, Peso was used to guide PEEP titration to achieve a positive end-expiratory P_TP_ of 0–2 cmH_2_O, preventing atelectasis without causing overdistension.Outcome: After neuromuscular blockade, the patient’s gas exchange stabilized, and inflammatory markers trended down. After 24 h, the infusion was stopped, and she was able to tolerate synchronized ventilation on minimal sedation. Teaching point: The set ΔP on the ventilator is meaningless in the face of vigorous patient effort. Recognizing and managing P-SILI, often requiring deep sedation and/or neuromuscular blockade in early severe ARDS, is essential for effective lung and RV protection. In selected patients, partial neuromuscular blockade titrated to respiratory drive—guided by respiratory rate and occlusion pressure—may serve as an intermediate strategy to maintain synchrony and minimize injurious effort without complete paralysis. **B. Phenotype paradigms: Extrapulmonary ARDS** ***Case 3: ARDSexp with intra-abdominal hypertension and preload limitation*** Patient presentation: A 45-year-old male develops distributive shock and ARDS (PaO_2_/FiO_2_ of 140) in the setting of severe acute pancreatitis. He has received 12 L of crystalloid and has tense abdominal distension. Physiological data:Ventilator: V_T_ 6 mL kg^−1^ PBW, PEEP 16 cmH_2_O, Pplat 35 cmH_2_O, ΔP 19 cmH_2_O.Hemodynamics: MAP 60 mmHg on high-dose norepinephrine and vasopressin. CVP 22 mmHg. Cardiac output is low.Monitoring: Bladder pressure is 25 mmHg (intra-abdominal hypertension grade III). Bedside echo shows a small, hyperdynamic left ventricle and a small, collapsible right ventricle. Esophageal manometry shows an end-expiratory Ppl of 20 cmH_2_O, yielding a P_TP_ of only 15 cmH_2_O.Clinical challenge: The high plateau pressure suggests severe lung injury, but the esophageal manometry and echo findings reveal the true problem: critically elevated Ppl from intra-abdominal hypertension is impeding venous return, causing preload-dependent shock despite a high CVP reading. The high PEEP is exacerbating this by further increasing intrathoracic pressure. Management and rationale:Reduce intra-abdominal pressure: An urgent percutaneous catheter was placed by interventional radiology to drain 3 L of ascetic fluid. This is the primary therapy to address the source of the high Ppl.Optimize ventilator for preload: PEEP was immediately reduced from 16 to 10 cmH_2_O. The goal was to lower the intrathoracic pressure to improve the pressure gradient for venous return. The low P_TP_ indicated that high PEEP was not necessary for alveolar stability and was primarily causing hemodynamic harm.Guided resuscitation: Despite the high CVP, the echo findings suggested preload limitation. After abdominal decompression, a passive leg raise maneuver was performed, which showed a 20% increase in cardiac output, confirming fluid responsiveness. A small fluid bolus was cautiously administered.Outcome: Following abdominal decompression and ventilator adjustment, the patient’s MAP stabilized, and vasopressor requirements decreased significantly. Teaching point: In ARDSexp driven by intra-abdominal hypertension, airway pressures are dangerously misleading. The pathophysiology is preload-dominant circulatory failure, not primarily lung stiffness. Treatment must focus on reducing abdominal/pleural pressure and restoring venous return. ***Case 4: ARDSexp in a morbidly obese patient*** Patient presentation: A 68-year-old female (BMI 52 kg m^−2^) with urosepsis develops ARDS. She is difficult to oxygenate, and the team is hesitant to increase PEEP due to high plateau pressures. Physiological data:Ventilator: V_T_ 6 mL kg^−1^ PBW, PEEP 10 cmH_2_O, Pplat 38 cmH_2_O, ΔP 28 cmH_2_O.Hemodynamics: MAP 68 mmHg on moderate-dose norepinephrine.Monitoring: Esophageal manometry reveals a high end-expiratory Ppl of 22 cmH_2_O due to massive chest wall weight. The end-expiratory P_TP_ is −12 cmH_2_O, indicating extensive atelectasis.Clinical challenge: The extremely high plateau and ΔP are driven almost entirely by the stiff, heavy chest wall, not by stiff lungs. The negative P_TP_ confirms that the current PEEP level is insufficient to keep the dependent lung open, leading to refractory hypoxemia from shunt. Management and rationale:Peso-guided PEEP titration: A PEEP titration was performed with the goal of achieving a positive end-expiratory P_TP_ of 0–2 cmH_2_O to overcome the Ppl and recruit the collapsed lung. This required increasing PEEP to 24 cmH_2_O.Monitor hemodynamics and P_TP_: With the PEEP increase, the Pplat rose to 45 cmH_2_O. However, the end-inspiratory P_TP_ remained acceptable at 21 cmH_2_O, and crucially, cardiac output did not fall, indicating the right ventricle was tolerating the intervention because the PEEP was recruiting lung rather than overdistending it.Reverse Trendelenburg positioning: The patient was placed in a reverse Trendelenburg position to help displace the abdominal contents caudally, improving diaphragmatic excursion and C_CW_.Outcome: Oxygenation improved dramatically (PaO_2_/FiO_2_ > 200), and the ΔP calculated from P_TP_ (P_TPinsp_ − P_TPexp_) was only 14 cmH_2_O. Teaching point: In morbid obesity, plateau pressure is a poor surrogate for lung stress. Esophageal manometry is invaluable for safely applying the high levels of PEEP needed to overcome severe chest wall elastance and achieve lung recruitment without causing hemodynamic collapse. **C. Phenotype paradigms: Mixed/alternating ARDS** ***Case 5: Evolving phenotype (ARDSexp → ARDSp)*** Patient presentation: A 70-year-old male with perforated diverticulitis (ARDSexp) is managed for 5 days. Initially, his hemodynamics were preload-dependent. On day 6, he develops a fever, new infiltrates on chest X-ray, and worsening hypoxemia. Physiological data:Initial (Day 2): PEEP 14 cmH_2_O, Pplat 32 cmH_2_O, high Ppl, small right ventricle. Managed with moderate PEEP and fluid optimization.Current (Day 6): PEEP 14 cmH_2_O, Pplat 32 cmH_2_O, but now the echo shows new, severe RV dilation and dysfunction. The CVP is rising. A diagnosis of ventilator-associated pneumonia (VAP) is made.Clinical challenge: The patient has evolved from a preload-limited ARDSexp phenotype to a mixed phenotype with a new, superimposed ARDSp component from the VAP. The ventilator strategy that was previously appropriate is now causing RV failure because the newly consolidated lung has made the patient highly sensitive to afterload. Management and rationale:Re-evaluate and adapt strategy: Recognizing the phenotypic shift is the critical first step. The management plan was pivoted from prioritizing venous return to prioritizing RV unloading.Reduce ΔP: The ΔP, which had been tolerated before, was now likely injurious. V_T_ was reduced to lower the ΔP from 18 to 14 cmH_2_O.Initiate prone positioning: Prone positioning, which may not have been a priority during the pure ARDSexp phase, was now initiated to recruit the new dorsal consolidation from the VAP and unload the acutely strained right ventricle.Outcome: The patient’s RV function improved over the next 12 h. Teaching point: ARDS is not a static disease. Clinicians must be prepared for the patient’s phenotype to evolve (e.g., after a secondary insult like VAP) and must continuously re-evaluate physiology to adapt the ventilator and hemodynamic strategy accordingly. ***Case 6: The “dual-hit” therapeutic paradox*** Patient presentation: A 55-year-old with severe COVID-19 pneumonia (ARDSp) develops septic shock. Due to profound capillary leak and vasoplegia, he receives massive volume resuscitation, leading to anasarca and a tense, fluid-overloaded abdominal and thoracic cavity. Physiological data:Ventilator: V_T_ 6 mL kg^−1^, PEEP 12 cmH_2_O, Pplat 35 cmH_2_O, ΔP 23 cmH_2_O.Hemodynamics: Refractory shock requiring three vasopressors.Monitoring: Pulmonary artery catheter shows high PVR and low cardiac index. Esophageal manometry shows high Ppl. Echo shows a dilated, failing right ventricle and an underfilled left ventricle.Clinical challenge: This patient represents a profound therapeutic paradox. He has a stiff, poorly recruitable lung (ARDSp) that makes his right ventricle exquisitely sensitive to afterload increases from PEEP/ΔP. Simultaneously, he has a fluid-overloaded, stiff chest wall (ARDSexp features) that is causing high Ppl and impeding venous return. Increasing PEEP to recruit the lung will worsen RV failure and preload limitation. Decreasing PEEP may worsen hypoxemia. Management and rationale:Recognize futility of conventional escalation: The team recognized that further increases in PEEP or vasopressors were likely to be futile and harmful.Ultra-protective ventilation and RV unloading: The decision was made to pursue VV-ECMO. This was chosen not just for refractory hypoxemia, but as the most potent therapy to unload the right ventricle.ECMO-facilitated strategy: Once on VV-ECMO, the ventilator settings were reduced to “rest” settings (ΔP near zero). This minimized airway pressures, profoundly reducing RV afterload. The ECMO support also allowed for aggressive diuresis to reduce total body water and improve C_CW_.Outcome: On VV-ECMO, the patient’s RV function recovered, and vasopressor requirements decreased. He was eventually liberated from all support. Teaching point: The “dual-hit” mixed phenotype, with both stiff lungs and a stiff chest wall, represents the limit of conventional management. Early consideration of VV-ECMO as an RV-unloading and lung-resting strategy can be lifesaving. ***Case 7: Distributive-cardiogenic overlap phenotype*** Patient presentation: A 76-year-old with a history of heart failure with preserved ejection fraction (HFpEF) and hypertension is admitted with pneumonia, septic shock, and ARDS. Physiological data:Ventilator: V_T_ 6 mL kg^−1^, PEEP 10 cmH_2_O, ΔP 14 cmH_2_O.Hemodynamics: MAP 65 mmHg on norepinephrine, lactate 3.8 mmol L^−1^, CI 1.8 L min^−1^ m^−2^.Monitoring: Echo shows global LV hypokinesis with an EF of 25–30% and evidence of diastolic dysfunction, alongside moderate RV dysfunction without severe dilation.Clinical challenge: This patient has biventricular myocardial depression in the setting of vasoplegia. The therapeutic challenge is to support systemic perfusion without exacerbating either RV or LV failure. Vasoconstriction from norepinephrine could worsen RV afterload, while excessive PEEP could strain the right ventricle. Inadequate PEEP could lead to hypoxemia and atelectasis, stressing both ventricles. Management and rationale:Combined inotropy and vasopressor support: Dobutamine was started at a low dose and carefully titrated to improve myocardial contractility and cardiac output. Early vasopressin administration enabled lowering the norepinephrine dose; both vasopressors ensured adequate systemic perfusion pressure for the vital organs and coronary arteries without increasing PVR.Balanced ventilator strategy: The ventilator was managed to avoid physiological extremes. Increases in PEEP were avoided to prevent any further increase in RV afterload. Permissive hypercapnia was limited, with a goal pH > 7.30 to avoid acidosis-induced pulmonary vasoconstriction.Judicious fluid management: A strategy of de-resuscitation with diuretics was initiated early, guided by daily weight and echo assessment of filling pressures, to reduce both pulmonary and systemic venous congestion.Outcome: With balanced hemodynamic support and careful ventilation, the cardiac index improved and lactate cleared. Teaching point: When distributive shock and ARDS coexist with pre-existing or sepsis-induced biventricular failure, therapy must be a careful balancing act. A combined vasopressor/inotrope strategy is often necessary, and the ventilator must be titrated to minimize stressors on both ventricles simultaneously.

## Figures and Tables

**Table 1:** ARDS phenotypes: mechanics and hemodynamic implications.

Feature	ARDSp	ARDSexp	Mixed/Alternative ARDS
Common causes	Pneumonia, aspiration, contusion	Abdominal infection, pancreatitis, transfusion, trauma	Features of both phenotypes; variable mechanics depending on dominant injury pattern
Primary injury site	Alveolar epithelium	Vascular endothelium	Features of both phenotypes; variable mechanics depending on dominant injury pattern
Imaging	Dense consolidation in dependent lobes	Diffuse atelectasis/interstitial edema	Features of both phenotypes; variable mechanics depending on dominant injury pattern
Lung compliance	Markedly reduced	Often relatively preserved	Features of both phenotypes; variable mechanics depending on dominant injury pattern
Chest wall compliance	Usually near-normal	Reduced (obesity, ascites, tense abdomen)	Features of both phenotypes; variable mechanics depending on dominant injury pattern
Pleural pressure	Normal or modestly increased	Often markedly increased	Features of both phenotypes; variable mechanics depending on dominant injury pattern
Transpulmonary pressure	High for a given Pplat	Lower for a given Pplat	Features of both phenotypes; variable mechanics depending on dominant injury pattern
Recruitability	Generally lower	Often higher	Features of both phenotypes; variable mechanics depending on dominant injury pattern
RV afterload	Increased (↑P_TP_, ↑PVR)	Lower/unchanged (↓P_TP_)	Features of both phenotypes; variable mechanics depending on dominant injury pattern
Venous return	Preserved or reduced if right ventricle fails	Reduced by ↑Ppl (preload-limited)	Features of both phenotypes; variable mechanics depending on dominant injury pattern
Management emphasis	Limit P_TP_/ΔP; prone; avoid excessive PEEP	Manage abdominal pressure; P_TP_-guided PEEP; restore venous return	Features of both phenotypes; variable mechanics depending on dominant injury pattern

ARDSp, pulmonary ARDS; ARDSexp, extrapulmonary ARDS; Pplat, plateau pressure; RV, right ventricular; P_TP_, transpulmonary pressure; PVR, pulmonary vascular resistance; ΔP, driving pressure; PEEP, positive end-expiratory pressure.

**Table 2:** Practical application of the pulmonary artery catheter in combined distributive shock and ARDS.

Parameter	Physiological Meaning	Pattern/Threshold	Likely Pathophysiology	Guided Intervention
P_RA_ (CVP)	Venous return (RV preload)	↑ P_RA_ + ↓ CO	RV failure or excessive PEEP	Reduce ΔP/PEEP; consider vasodilator
mPAP	RV afterload	↑ mPAP + normal PCWP	Alveolar overdistension, high PVR	Decrease P_TP_; prone; pulmonary vasodilator
PCWP	LV filling pressure	↑ PCWP + ↑ P_RA_	Biventricular depression, excessive fluids	Restrict fluids; add inotrope
CO/CI	Global flow	↓ CI < 2.2 L min^−1^ m^−2^	RV or LV dysfunction	Optimize preload; consider inotrope
PVR	Steady afterload	>240 dynes sec cm^−5^ (≈3 Wood units)	Vascular compression, hypoxia, microthrombosis	Optimize oxygenation; vasodilator therapy
Cpa	Pulsatile afterload	↓ <2 mL mmHg^−1^	Stiff pulmonary vasculature	Reduce ΔP; prone; inodilator
SvO_2_	Global oxygen balance	↓ <60%	Imbalance between DO_2_/VO_2_; early RV uncoupling	Optimize CO, Hb, FiO_2_
DPG	Microvascular obstruction	>7 mmHg	Pulmonary vasculopathy	Consider selective vasodilators

P_RA_, right atrial pressure; RV, right ventricular; CO, cardiac output; PEEP, positive end-expiratory pressure; ΔP, driving pressure; mPAP, mean pulmonary artery pressure; PCWP, pulmonary capillary wedge pressure; PVR, pulmonary vascular resistance; P_TP_, transpulmonary pressure; LV, left ventricular; CI, cardiac index; Cpa, pulmonary artery compliance; SvO_2_, mixed venous oxygen saturation; DO_2_, oxygen delivery; VO_2_, oxygen consumption; Hb, hemoglobin; FiO_2_, fraction of inspired oxygen. DPG, diastolic pulmonary gradient.

**Table 3:** Hemodynamic phenotype-directed vasoactive strategy.

Phenotype	Primary Problem	First-Line	Adjuncts	Cautions
Vasoplegia-predominant	Low SVR, adequate output	Norepinephrine	Vasopressin	Avoid excessive ΔP raising PVR
RV overload (ARDSp)	High P_TP_/PVR → RV failure	Lower ΔP; optimize PEEP; prone	Inotrope/inodilator (dobutamine/milrinone); inhaled vasodilator	Avoid pure vasoconstrictors that raise PAP
Preload-limited (high Ppl/IAP; ARDSexp)	Venous return impeded	Reduce PEEP; relieve IAP	Gentle fluids; venoconstriction	Beware fluid overload and worsening oxygenation
Mixed/alternative ARDS (ARDSp ↔ ARDSexp)	Features of both phenotypes; variable mechanics depending on dominant injury pattern	Tailored combination of pulmonary and extrapulmonary strategies	Adjust ventilator and vasoactive settings dynamically as dominant feature shifts	Monitor for both RV overload and preload limitation
Biventricular depression	Low contractility both ventricles	Norepinephrine and inotrope/inodilator	Consider pulmonary vasodilation	Monitor for hypotension with inodilators

SVR, systemic vascular resistance; ΔP, driving pressure; PVR, pulmonary vascular resistance; ARDSp, pulmonary ARDS; P_TP_, transpulmonary pressure; RV, right ventricular; PEEP, positive end-expiratory pressure; PAP, pulmonary artery pressure; Ppl, pleural pressure; IAP, intra-abdominal pressure; ARDSexp, extrapulmonary ARDS.

**Table 4:** Practical ventilator titration in ARDS with distributive shock.

Target/Constraint	Rationale	ARDSp (RV-Overload-Prone)	ARDSexp (Preload-Limited)	Mixed/Alternative ARDS
V_T_ 4–6 mL kg^−1^ PBW	Prevent overdistension	Aim 4–5 mL kg^−1^ if RV strain	5–6 mL kg^−1^ with P_TP_ guidance	Features of both phenotypes; variable mechanics depending on dominant injury pattern
Pplat ≤ 30 cmH_2_O	Lung protection	Keep lower if high P_TP_	Interpret with Peso; may tolerate higher Pplat if low P_TP_	Features of both phenotypes; variable mechanics depending on dominant injury pattern
ΔP minimization	Surrogate of strain	Reduce via V_T_/PEEP trade-off	Reduce via recruitability-guided PEEP	Features of both phenotypes; variable mechanics depending on dominant injury pattern
PEEP strategy	Balance recruitment vs. RV load	Favor moderate PEEP; avoid overdistension	Use P_TP_ targets; manage IAP first	Features of both phenotypes; variable mechanics depending on dominant injury pattern
Permissive hypercapnia	Avoid ↑PVR	Maintain pH ≥ 7.25 (buffer if needed)	Similar, but avoid severe hypercapnia if PAP rise	Features of both phenotypes; variable mechanics depending on dominant injury pattern
Prone positioning	Oxygenation, RV unloading	Early and prolonged	Consider especially if dorsal atelectasis prominent	Features of both phenotypes; variable mechanics depending on dominant injury pattern
Inhaled vasodilators	RV/PVR rescue	Consider NO/epoprostenol as bridge	Consider for transient mismatch during repositioning	Features of both phenotypes; variable mechanics depending on dominant injury pattern
Weaning cues	Avoid derecruitment	Decrease PEEP after RV recovery	Reassess Peso/IAP; ensure venous return stable	Features of both phenotypes; variable mechanics depending on dominant injury pattern

ARDSp, pulmonary ARDS; RV, right ventricular; ARDSexp, extrapulmonary ARDS; V_T_, tidal volume; P_TP_, transpulmonary pressure; Pplat, plateau pressure; Peso, esophageal pressure; ΔP, driving pressure; PEEP, positive end-expiratory pressure; IAP, intra-abdominal pressure; PVR, pulmonary vascular resistance; PAP, pulmonary artery pressure; NO, nitric oxide.

**Table 5:** Monitoring matrix.

Clinical Question	Best Tool	Secondary Tool	Pitfalls	Mixed/Alternative ARDS Considerations
Is P_TP_ excessive?	Esophageal pressure	ΔP trends	Peso artifacts; patient effort	May vary by region; combine with imaging and recruitment testing
Is venous return limited by Ppl?	Echo IVC/RV size; Peso	Venous excess ultrasound	CVP misleads when Ppl high	Mixed phenotype may have variable preload limitation
Is RV-PA uncoupled?	Echo (RV size/TAPSE/PASP)	PA catheter (PVR, SvO_2_)	Ignoring LV-RV interdependence	Changes over course; reassess frequently
Fluid responsive?	PLR with echo VTI	PPV, SVV (adjusted for PVR)	False positives with high PVR	Mixed pattern may respond differently depending on dominant physiology
Flow vs. content limiting DO_2_?	SvO_2_/ScvO_2_ and lactate	NIRS	Unreliable if severe anemia or microcirculatory failure	Monitor both macro and micro hemodynamics closely

P_TP_, transpulmonary pressure; ΔP, driving pressure; Ppl, pleural pressure; IVC, inferior vena cava; RV, right ventricular; Peso, esophageal pressure; CVP, central venous pressure; RV-PA, right ventricular-pulmonary arterial; TAPSE, tricuspid annular plane systolic excursion; PASP, pulmonary artery systolic pressure; PA, pulmonary catheter; PVR, pulmonary vascular resistance; SvO_2_, mixed venous oxygen saturation, LV-RV, left ventricular-right ventricular; PLR, passive leg raising; VTI, velocity time integral; PPV, pulse pressure variation; SVV, stroke volume variation; DO_2_, oxygen delivery; ScvO_2_, central venous oxygen saturation; NIRS, near-infrared spectroscopy.

**Table 6:** A paradigm of heart–lung interaction implementation checklist for the first 6 h.

1. Confirm ARDS and grade hypoxemia; exclude hydrostatic edema
2. Classify phenotype: ARDSp vs. ARDSexp vs. mixed/alternative ARDS based on history, imaging, and mechanics
3. Initiate lung-protective ventilation (V_T_ 4–6 mL kg^−1^ predicted body weight; aim for lowest feasible ΔP); set initial PEEP using recruitability and, when available, Peso-estimated P_TP_
4. Perform focused echocardiography to characterize RV size/function and LV filling
5. Start norepinephrine to target MAP ≥ 65 mmHg; add vasopressin for catecholamine sparing if vasoplegia predominates and/or to decrease PVR
6. Decide on fluid strategy using PLR/echo; avoid indiscriminate boluses; consider early conservative strategy
7. Evaluate for proning within the first 12–24 h in moderate-to-severe ARDS; prepare team and checklists
8. If RV strain is present, avoid excessive PEEP, buffer acidosis, and consider inhaled vasodilator as a bridge
9. Seek and control the infectious source; start empiric antimicrobials based on local ecology and de-escalate with culture data
10. Reassess after each intervention—if hypotension worsens with increased PEEP, suspect RV afterload rise

ARDSp, pulmonary ARDS; ARDSexp, extrapulmonary ARDS; V_T_, tidal volume; ΔP, driving pressure; PEEP, positive end-expiratory pressure; Peso, esophageal pressure; P_TP_, transpulmonary pressure; RV, right ventricular; LV, left ventricular; MAP, mean arterial pressure; PVR, pulmonary vascular resistance; PLR, passive leg raising.

**Table 7:** Educational pitfalls and how to avoid them.

**Pitfall 1:** Equating P_PLAT_ pressure with lung stress. **Reality:** P_PLAT_ combines lung and chest wall components; without P_TP_, identical P_PLAT_ can represent very different alveolar stresses.
**Pitfall 2:** Chasing oxygenation with ever-higher PEEP in ARDSp. **Reality:** Above the individual’s closing pressure, further PEEP may simply raise P_TP_ and PVR, precipitating RV failure.
**Pitfall 3:** Ignoring IAP and body habitus. **Reality:** Elevated IAP reduces venous return and inflates Ppl; the fix is not always more fluid or more PEEP.
**Pitfall 4:** Treating all hypotension in ARDS as vasoplegia. **Reality:** RV failure can mimic vasoplegia. A falling MAP with rising CVP, visible RV dilation, and decreasing LV filling suggests RV-afterload crisis, not simply low SVR.
**Pitfall 5:** Over-interpreting pulse pressure variation in high PVR. **Reality:** Pulse pressure variation may be large because of RV-PA uncoupling rather than preload responsiveness.
**Pitfall 6:** Under-using prone positioning. **Reality:** Prone improves oxygenation and often RV performance; it should not be reserved solely for refractory hypoxemia.
**Pitfall 7:** Forgetting the venous side. **Reality:** Restoring stressed volume with vasopressors and positioning may be more effective than large fluid boluses in distributive physiology.

P_PLAT_, plateau pressure; P_TP_, transpulmonary pressure; PEEP, positive end-expiratory pressure; ARDSp, pulmonary ARDS; PVR, pulmonary vascular resistance RV, right ventricular; IAP, intra-abdominal pressure; Ppl, pleural pressure; MAP, mean arterial pressure, CVP, central venous pressure; LV, left ventricular; SVR, systemic vascular resistance; RV-PA, right ventricular-pulmonary arterial.

## Data Availability

No new data were created.

## Abbreviations

The following abbreviations are used in this manuscript:

ARDS	Acute respiratory distress syndrome
ICU	Intensive care unit
PA	Pulmonary artery
PAC	Pulmonary artery catheter
RV	Right ventricular
PEEP	Positive end-expiratory pressure
ΔP	Driving pressure
PTP	Transpulmonary pressure
SVR	Systemic vascular resistance
PRA	Right atrial pressure
Paw	Alveolar (airway) pressure
PVR	Pulmonary vascular resistance
Ppl	Pleural pressure
ARDSp	Pulmonary acute respiratory distress syndrome
ARDSexp	Extrapulmonary acute respiratory distress syndrome
CL	Compliance of the lung
CCW	Compliance of the chest wall
LV	Left ventricular
Ea	Effective arterial elastance
Ees	End-systolic elastance
ESPVR	End-systolic pressure-volume relationship
SV	Stroke volume
Cpa	Pulmonary artery compliance
Peso	Esophageal pressure
PAP	Pulmonary artery pressure
mPAP	Mean pulmonary artery pressure
PCWP	Pulmonary capillary wedge pressure
CVP	Central venous pressure
TAPSE	Tricuspid annular plane systolic excursion
FAC	Fractional area change
LVOT VTI	Left ventricular outflow tract velocity time integral
TPG	Transpulmonary gradient
DPG	Diastolic pulmonary gradient
SvO2	Mixed venous oxygen saturation
SVV	Stroke volume variation
PPV	Pulse pressure variation
MAP	Mean arterial pressure
Eadyn	Dynamic arterial elastance
PaO2	Arterial partial pressure of oxygen
V/Q	Ventilation/perfusion
FiO2	Fraction of inspired oxygen
ECMO	Extracorporeal membrane oxygenation
VV	Veno-venous
VA	Veno-arterial
ECCO2R	Extracorporeal carbon dioxide removal
P-SILI	Patient self-inflicted lung injury
SBT	Spontaneous breathing trial

## References

[1] Bellani G., Laffey J.G., Pham T., Fan E., Brochard L., Esteban A., Gattinoni L., van Haren F., Larsson A., McAuley D.F. (2016). Epidemiology, Patterns of Care, and Mortality for Patients with Acute Respiratory Distress Syndrome in Intensive Care Units in 50 Countries. JAMA.

[2] Singer M., Deutschman C.S., Seymour C.W., Shankar-Hari M., Annane D., Bauer M., Bellomo R., Bernard G.R., Chiche J.D., Coopersmith C.M. (2016). The Third International Consensus Definitions for Sepsis and Septic Shock (Sepsis-3). JAMA.

[3] Rudd K.E., Johnson S.C., Agesa K.M., Shackelford K.A., Tsoi D., Kievlan D.R., Colombara D.V., Ikuta K.S., Kissoon N., Finfer S. (2020). Global, regional, and national sepsis incidence and mortality, 1990–2017: Analysis for the Global Burden of Disease Study. Lancet.

[4] Brower R.G., Matthay M.A., Morris A., Schoenfeld D., Thompson B.T., Wheeler A., Acute Respiratory Distress Syndrome Network (2000). Ventilation with lower tidal volumes as compared with traditional tidal volumes for acute lung injury and the acute respiratory distress syndrome. N. Engl. J. Med..

[5] Pinsky M.R. (2012). Heart lung interactions during mechanical ventilation. Curr. Opin. Crit. Care.

[6] Wheeler A.P., Bernard G.R., Thompson B.T., Schoenfeld D., Wiedemann H.P., deBoisblanc B., Connors A.F., Hite R.D., Harabin A.L., National Heart, Lung, and Blood Institute Acute Respiratory Distress Syndrome (ARDS) Clinical Trials Network (2006). Pulmonary-artery versus central venous catheter to guide treatment of acute lung injury. N. Engl. J. Med..

[7] Guérin C., Papazian L., Reignier J., Ayzac L., Loundou A., Forel J.M., Investigators of the Acurasys and Proseva Trials (2016). Effect of driving pressure on mortality in ARDS patients during lung protective mechanical ventilation in two randomized controlled trials. Crit. Care.

[8] Guarracino F., Bertini P., Pinsky M.R. (2019). Cardiovascular determinants of resuscitation from sepsis and septic shock. Crit. Care.

[9] Amato M.B., Meade M.O., Slutsky A.S., Brochard L., Costa E.L., Schoenfeld D.A., Stewart T.E., Briel M., Talmor D., Mercat A. (2015). Driving pressure and survival in the acute respiratory distress syndrome. N. Engl. J. Med..

[10] Mekontso Dessap A., Boissier F., Charron C., Bégot E., Repessé X., Legras A., Brun-Buisson C., Vignon P., Vieillard-Baron A. (2016). Acute cor pulmonale during protective ventilation for acute respiratory distress syndrome: Prevalence, predictors, and clinical impact. Intensive Care Med..

[11] Gattinoni L., Tonetti T., Cressoni M., Cadringher P., Herrmann P., Moerer O., Protti A., Gotti M., Chiurazzi C., Carlesso E. (2016). Ventilator-related causes of lung injury: The mechanical power. Intensive Care Med..

[12] Serpa Neto A., Deliberato R.O., Johnson A.E.W., Bos L.D., Amorim P., Pereira S.M., Cazati D.C., Cordioli R.L., Correa T.D., Pollard T.J. (2018). Mechanical power of ventilation is associated with mortality in critically ill patients: An analysis of patients in two observational cohorts. Intensive Care Med..

[13] Magder S. (2012). Bench-to-bedside review: An approach to hemodynamic monitoring–Guyton at the bedside. Crit. Care.

[14] Myburgh J.A. (2006). An appraisal of selection and use of catecholamines in septic shock-old becomes new again. Crit. Care Resusc..

[15] Cherpanath T.G., Lagrand W.K., Schultz M.J., Groeneveld A.B. (2013). Cardiopulmonary interactions during mechanical ventilation in critically ill patients. Neth. Heart J..

[16] Yuriditsky E., Mireles-Cabodevila E., Alviar C.L. (2025). How I Teach: Heart-Lung Interactions during Mechanical Ventilation. Positive Pressure and the Right Ventricle. ATS Sch..

[17] Kenny J.E. (2020). An Approach to Mechanical Heart-Lung Interaction.

[18] Repessé X., Vieillard-Baron A. (2017). Right heart function during acute respiratory distress syndrome. Ann. Transl. Med..

[19] Wilkman E., Kuitunen A., Pettilä V., Varpula M. (2014). Fluid responsiveness predicted by elevation of PEEP in patients with septic shock. Acta Anaesthesiol. Scand..

[20] Gattinoni L., Pelosi P., Suter P.M., Pedoto A., Vercesi P., Lissoni A. (1998). Acute respiratory distress syndrome caused by pulmonary and extrapulmonary disease. Different syndromes?. Am. J. Respir. Crit. Care Med..

[21] Gattinoni L., Chiumello D., Carlesso E., Valenza F. (2004). Bench-to-bedside review: Chest wall elastance in acute lung injury/acute respiratory distress syndrome patients. Crit. Care.

[22] Akoumianaki E., Maggiore S.M., Valenza F., Bellani G., Jubran A., Loring S.H., Pelosi P., Talmor D., Grasso S., Chiumello D. (2014). The application of esophageal pressure measurement in patients with respiratory failure. Am. J. Respir. Crit. Care Med..

[23] Repessé X., Vieillard-Baron A., Geri G. (2018). Value of measuring esophageal pressure to evaluate heart-lung interactions-applications for invasive hemodynamic monitoring. Ann. Transl. Med..

[24] Bentley R.F., Yo S.W., Mok K.H., Valle F.H., Goligher E.C., Carvalho C.G., Granton J.T., Mak S.S., Ryan C.M. (2025). Impact of positive airway pressure on right ventricular afterload in pulmonary arterial hypertension. ERJ Open Res..

[25] Mesquida J., Kim H.K., Pinsky M.R. (2011). Effect of tidal volume, intrathoracic pressure, and cardiac contractility on variations in pulse pressure, stroke volume, and intrathoracic blood volume. Intensive Care Med..

[26] O’Quin R.J., Marini J.J., Culver B.H., Butler J. (1985). Transmission of airway pressure to pleural space during lung edema and chest wall restriction. J. Appl. Physiol..

[27] Lansdorp B., Hofhuizen C., van Lavieren M., van Swieten H., Lemson J., van Putten M.J., van der Hoeven J.G., Pickkers P. (2014). Mechanical ventilation-induced intrathoracic pressure distribution and heart-lung interactions. Crit. Care Med..

[28] Pinsky M.R. (2014). Why knowing the effects of positive-pressure ventilation on venous, pleural, and pericardial pressures is important to the bedside clinician?. Crit. Care Med.

[29] Corp A., Thomas C., Adlam M. (2021). The cardiovascular effects of positive pressure ventilation. BJA Educ..

[30] Pinsky M.R., Matuschak G.M., Klain M. (1985). Determinants of cardiac augmentation by elevations in intrathoracic pressure. J. Appl. Physiol..

[31] Matuschak G.M., Pinsky M.R., Klain M. (1986). Hemodynamic effects of synchronous high-frequency jet ventilation during acute hypovolemia. J. Appl. Physiol..

[32] Pinsky M.R., Matuschak G.M., Bernardi L., Klain M. (1986). Hemodynamic effects of cardiac cycle-specific increases in intrathoracic pressure. J. Appl. Physiol..

[33] Fraile-Gutiérrez V., Zapata-Fenor L., Blandino-Ortiz A., Guerrero-Mier M., Ochagavia-Calvo A. (2024). Right ventricular dysfunction in the critically ill. Echocardiographic evaluation. Med. Intensiva (Engl. Ed.).

[34] Bowcock E., Huang S., Yeo R., Walisundara D., Duncan C.F., Pathan F., Strange G., Playford D., Orde S. (2024). The value of right ventricular to pulmonary arterial coupling in the critically ill: A National Echocardiography Database of Australia (NEDA) substudy. Ann. Intensive Care.

[35] Bastos M.B., Burkhoff D., Maly J., Daemen J., den Uil C.A., Ameloot K., Lenzen M., Mahfoud F., Zijlstra F., Schreuder J.J. (2020). Invasive left ventricle pressure-volume analysis: Overview and practical clinical implications. Eur. Heart J..

[36] Brener M.I., Masoumi A., Ng V.G., Tello K., Bastos M.B., Cornwell W.K., Hsu S., Tedford R.J., Lurz P., Rommel K.P. (2022). Invasive Right Ventricular Pressure-Volume Analysis: Basic Principles, Clinical Applications, and Practical Recommendations. Circ. Heart Fail..

[37] Yao M., Wu Z., Zhang L., Ji M., Qin S., He Q., Lin Y., Xie M., Li Y. (2025). Clinical Usefulness of Right Ventricular-Pulmonary Artery Coupling in Patients with Heart Failure. Diagnostics.

[38] Ma Q., Ding C., Wei W., Su C., Li B., Zhou Z., Chen C., Liu B., Zhang X., Wu J. (2024). The value of right ventricular pulmonary artery coupling in determining the prognosis of patients with sepsis. Sci. Rep..

[39] Kelly R.P., Ting C.T., Yang T.M., Liu C.P., Maughan W.L., Chang M.S., Kass D.A. (1992). Effective arterial elastance as index of arterial vascular load in humans. Circulation.

[40] Chemla D., Lau E.M., Papelier Y., Attal P., Hervé P. (2015). Pulmonary vascular resistance and compliance relationship in pulmonary hypertension. Eur. Respir. J..

[41] Pinsky M.R. (2016). The right ventricle: Interaction with the pulmonary circulation. Crit. Care.

[42] Guinot P.G., Andrei S., Longrois D. (2022). Ventriculo-arterial coupling: From physiological concept to clinical application in peri-operative care and ICUs. Eur. J. Anaesthesiol. Intensive Care.

[43] Zochios V., Parhar K., Tunnicliffe W., Roscoe A., Gao F. (2017). The Right Ventricle in ARDS. Chest.

[44] Hollenberg S.M. (2025). Sepsis-Associated Cardiomyopathy: Long-Term Prognosis, Management, and Guideline-Directed Medical Therapy. Curr. Cardiol. Rep..

[45] Martin L., Derwall M., Al Zoubi S., Zechendorf E., Reuter D.A., Thiemermann C., Schuerholz T. (2019). The Septic Heart: Current Understanding of Molecular Mechanisms and Clinical Implications. Chest.

[46] Ganeriwal S., Alves Dos Anjos G., Schleicher M., Hockstein M.A., Tonelli A.R., Duggal A., Siuba M.T. (2023). Right ventricle-specific therapies in acute respiratory distress syndrome: A scoping review. Crit. Care.

[47] Spinelli E., Scaramuzzo G., Slobod D., Mauri T. (2023). Understanding cardiopulmonary interactions through esophageal pressure monitoring. Front. Physiol..

[48] Vignon P. (2023). Cardiopulmonary interactions during ventilator weaning. Front. Physiol..

[49] Petit M., Vieillard-Baron A. (2023). Ventricular interdependence in critically ill patients: From physiology to bedside. Front. Physiol..

[50] Santamore W.P., Gray L. (1995). Significant left ventricular contributions to right ventricular systolic function. Mechanism and clinical implications. Chest.

[51] Gong S., Ding X., Wang X., Chinese Critical Ultrasound Study Group (CCUSG) (2024). Assessment of Pulmonary Circulation of Critically Ill Patients Based on Critical Care Ultrasound. J. Clin. Med..

[52] Rajaram S.S., Desai N.K., Kalra A., Gajera M., Cavanaugh S.K., Brampton W., Young D., Harvey S., Rowan K. (2013). Pulmonary artery catheters for adult patients in intensive care. Cochrane Database Syst. Rev..

[53] See K.C., Ng J., Siow W.T., Ong V., Phua J. (2017). Frequency and prognostic impact of basic critical care echocardiography abnormalities in patients with acute respiratory distress syndrome. Ann. Intensive Care.

[54] Tello K., Wan J., Dalmer A., Vanderpool R., Ghofrani H.A., Naeije R., Roller F., Mohajerani E., Seeger W., Herberg U. (2019). Validation of the Tricuspid Annular Plane Systolic Excursion/Systolic Pulmonary Artery Pressure Ratio for the Assessment of Right Ventricular-Arterial Coupling in Severe Pulmonary Hypertension. Circ. Cardiovasc. Imaging.

[55] Lang R.M., Badano L.P., Mor-Avi V., Afilalo J., Armstrong A., Ernande L., Flachskampf F.A., Foster E., Goldstein S.A., Kuznetsova T. (2015). Recommendations for cardiac chamber quantification by echocardiography in adults: An update from the American Society of Echocardiography and the European Association of Cardiovascular Imaging. Eur. Heart J. Cardiovasc. Imaging.

[56] Yastrebov K., Brunel L., Williams Z.A., Paterson H.S., Yata M., Burrows C.S., Wise I.K., Robinson B.M., Bannon P.G. (2020). Comparison of dynamic changes in stressed intravascular volume, mean systemic filling pressure and cardiovascular compliance: Pilot investigation and study protocol. PLoS ONE.

[57] Ball L., Talmor D., Pelosi P. (2024). Transpulmonary pressure monitoring in critically ill patients: Pros and cons. Crit. Care.

[58] Mauri T., Yoshida T., Bellani G., Goligher E.C., Carteaux G., Rittayamai N., Mojoli F., Chiumello D., Piquilloud L., Grasso S. (2016). Esophageal and transpulmonary pressure in the clinical setting: Meaning, usefulness and perspectives. Intensive Care Med..

[59] Repessé X., Charron C., Vieillard-Baron A. (2015). Acute cor pulmonale in ARDS: Rationale for protecting the right ventricle. Chest.

[60] Chiumello D., Fioccola A. (2024). Recent advances in cardiorespiratory monitoring in acute respiratory distress syndrome patients. J. Intensive Care.

[61] Cutuli S.L., Grieco D.L., Michi T., Cesarano M., Rosà T., Pintaudi G., Menga L.S., Ruggiero E., Giammatteo V., Bello G. (2023). Personalized Respiratory Support in ARDS: A Physiology-to-Bedside Review. J. Clin. Med..

[62] Baedorf Kassis E., Talmor D. (2021). Clinical application of esophageal manometry: How I do it. Crit. Care.

[63] Nieman G.F., Satalin J., Andrews P., Aiash H., Habashi N.M., Gatto L.A. (2017). Personalizing mechanical ventilation according to physiologic parameters to stabilize alveoli and minimize ventilator induced lung injury (VILI). Intensive Care Med. Exp..

[64] Costa E.L., Borges J.B., Melo A., Suarez-Sipmann F., Toufen C., Bohm S.H., Amato M.B. (2009). Bedside estimation of recruitable alveolar collapse and hyperdistension by electrical impedance tomography. Intensive Care Med..

[65] Gattinoni L., Pesenti A. (2005). The concept of “baby lung”. Intensive Care Med..

[66] Talmor D., Sarge T., Malhotra A., O’Donnell C.R., Ritz R., Lisbon A., Novack V., Loring S.H. (2008). Mechanical ventilation guided by esophageal pressure in acute lung injury. N. Engl. J. Med..

[67] Barrot L., Asfar P., Mauny F., Winiszewski H., Montini F., Badie J., Quenot J.P., Pili-Floury S., Bouhemad B., Louis G. (2020). LOCO_2_ Investigators and REVA Research Network. Liberal or Conservative Oxygen Therapy for Acute Respiratory Distress Syndrome. N. Engl. J. Med..

[68] ICU-ROX Investigators and the Australian and New Zealand Intensive Care Society Clinical Trials Group (2020). Conservative Oxygen Therapy during Mechanical Ventilation in the ICU. N. Engl. J. Med..

[69] Gendreau S., Geri G., Pham T., Vieillard-Baron A., Mekontso Dessap A. (2022). The role of acute hypercapnia on mortality and short-term physiology in patients mechanically ventilated for ARDS: A systematic review and meta-analysis. Intensive Care Med..

[70] Malbrain M.L.N.G., Van Regenmortel N., Saugel B., De Tavernier B., Van Gaal P.J., Joannes-Boyau O., Teboul J.L., Rice T.W., Mythen M., Monnet X. (2018). Principles of fluid management and stewardship in septic shock: It is time to consider the four D’s and the four phases of fluid therapy. Ann. Intensive Care.

[71] Alvarado Sánchez J.I., Caicedo Ruiz J.D., Diaztagle Fernández J.J., Amaya Zuñiga W.F., Ospina-Tascón G.A., Cruz Martínez L.E. (2021). Predictors of fluid responsiveness in critically ill patients mechanically ventilated at low tidal volumes: Systematic review and meta-analysis. Ann. Intensive Care.

[72] Myatra S.N., Monnet X., Teboul J.L. (2017). Use of ‘tidal volume challenge’ to improve the reliability of pulse pressure variation. Crit. Care.

[73] Monnet X., Marik P., Teboul J.L. (2016). Passive leg raising for predicting fluid responsiveness: A systematic review and meta-analysis. Intensive Care Med..

[74] Pesenti A., Slobod D., Magder S. (2023). The forgotten relevance of central venous pressure monitoring. Intensive Care Med..

[75] Magder S. (2017). Right Atrial Pressure in the Critically Ill: How to Measure, What Is the Value, What Are the Limitations?. Chest.

[76] Vieillard-Baron A., Matthay M., Teboul J.L., Bein T., Schultz M., Magder S., Marini J.J. (2016). Experts’ opinion on management of hemodynamics in ARDS patients: Focus on the effects of mechanical ventilation. Intensive Care Med..

[77] Evans L., Rhodes A., Alhazzani W., Antonelli M., Coopersmith C.M., French C., Machado F.R., Mcintyre L., Ostermann M., Prescott H.C. (2021). Surviving Sepsis Campaign: International Guidelines for Management of Sepsis and Septic Shock 2021. Crit. Care Med..

[78] Russell J.A. (2019). Vasopressor therapy in critically ill patients with shock. Intensive Care Med..

[79] Landry D.W., Levin H.R., Gallant E.M., Ashton R.C., Seo S., D’Alessandro D., Oz M.C., Oliver J.A. (1997). Vasopressin deficiency contributes to the vasodilation of septic shock. Circulation.

[80] Lajoye Q., Orieux A., Boyer A., Prevel R., Jozwiak M. (2025). Vasopressin and its analogues in patients with septic shock: Holy Grail or unfulfilled promise?. Crit. Care.

[81] Morelli A., Ertmer C., Rehberg S., Lange M., Orecchioni A., Laderchi A., Bachetoni A., D’Alessandro M., Van Aken H., Pietropaoli P. (2008). Phenylephrine versus norepinephrine for initial hemodynamic support of patients with septic shock: A randomized, controlled trial. Crit. Care.

[82] Krachman S.L., Lodato R.F., Morice R., Gutierrez G., Dantzker D.R. (1994). Effects of dobutamine on oxygen transport and consumption in the adult respiratory distress syndrome. Intensive Care Med..

[83] Guérin C., Reignier J., Richard J.C., Beuret P., Gacouin A., Boulain T., Mercier E., Badet M., Mercat A., Baudin O. (2013). Prone positioning in severe acute respiratory distress syndrome. N. Engl. J. Med..

[84] Vieillard-Baron A., Charron C., Caille V., Belliard G., Page B., Jardin F. (2007). Prone positioning unloads the right ventricle in severe ARDS. Chest.

[85] Peek G.J., Mugford M., Tiruvoipati R., Wilson A., Allen E., Thalanany M.M., Hibbert C.L., Truesdale A., Clemens F., Cooper N. (2009). Efficacy and economic assessment of conventional ventilatory support versus extracorporeal membrane oxygenation for severe adult respiratory failure (CESAR): A multicentre randomised controlled trial. Lancet.

[86] Combes A., Hajage D., Capellier G., Demoule A., Lavoué S., Guervilly C., Da Silva D., Zafrani L., Tirot P., Veber B. (2018). Extracorporeal Membrane Oxygenation for Severe Acute Respiratory Distress Syndrome. N. Engl. J. Med..

[87] Combes A., Supady A., Abrams D., Agerstrand C., Badulak J., Camporota L., Fan E., Ferguson N.D., Fraser J.F., Hodgson C. (2025). Extracorporeal life support for adult patients with ARDS. Intensive Care Med..

[88] Grant C., Richards J.B., Frakes M., Cohen J., Wilcox S.R. (2021). ECMO and Right Ventricular Failure: Review of the Literature. J. Intensive Care Med..

[89] Combes A., Fanelli V., Pham T., Ranieri V.M., European Society of Intensive Care Medicine Trials Group and the “Strategy of Ultra-Protective lung ventilation with Extracorporeal CO_2_ Removal for New-Onset moderate to severe ARDS” (SUPERNOVA) investigators (2019). Feasibility and safety of extracorporeal CO_2_ removal to enhance protective ventilation in acute respiratory distress syndrome: The SUPERNOVA study. Intensive Care Med..

[90] Sondergaard S. (2013). Pavane for a pulse pressure variation defunct. Crit. Care.

